# Implications of Pr^3+^ ions on structural, opto-thermal features of alkali zinc boro-tellurite glass systems for optical and laser technology applications

**DOI:** 10.1038/s41598-026-48568-2

**Published:** 2026-04-14

**Authors:** B. N. Shiva Kumar, C. Devaraja, G. V. Ashok Reddy, R. S. Gedam

**Affiliations:** 1 Department of Physics, Manipal Institute of Technology Bengaluru, Manipal Academy of Higher Education, Manipal, 576104 Karnataka India; 2 Department of Physics, Nitte Meenakshi Institute of Technology, Deemed to be University), Nitte, Bengaluru, 560064 Karnataka India; 3 Department of Physics, Visvesvaraya National Institute of Technology, Nagpur, 440010 Maharashtra India

**Keywords:** Boro-tellurite glass, Praseodymium, Refractive index, Metallization criterion, And Photoluminescence, Chemistry, Materials science, Optics and photonics, Physics

## Abstract

A novel multicomponent series of Pr^3+^ -ions doped borotellurite glasses with composition (45-y)B_2_O_3_+20TeO_2_+20ZnO+5Pb_3_O_4_+10Na_2_O+yPr_6_O_11_, where y = 0.0, 0.3, 0.6, 1.0, 1.5, and 2 mol%, were prepared. By the XRD technique, the non-crystalline state of the prepared glasses was validated. ATR-FTIR and Raman spectroscopy confirmed the presence of functional units, including BO_4_, BO_3_, TeO_4_, TeO_3_, and Pb–O and Zn–O links. The thermal analysis by DSC shows that glass transition and crystallization temperatures are found between 409 ${\rm ^\circ C}$ and 461 ${\rm ^\circ C}$, and 511 ${\rm ^\circ C}$ and 572 ${\rm ^\circ C}$. UV-visible absorption spectroscopy characterization reveals the transitions ^3^H_4_ → ^3^P_2_, ^3^P_1_, ^3^P_0_, and ^1^D_2_ corresponding to wavelengths of 444 nm, 470 nm, 483 nm, and 590 nm. The emission spectra of Pr^3+^ ions embedded in glass samples were recorded by a spectrofluorometer with an excitation wavelength of 444 nm. Among the observed transitions, the ^3^P_0_ → ^3^H_4_ (488 nm) and ^3^P_1_ → ^3^H_5_ (605 nm) transitions exhibited the most intense peaks. The CCT value of orange emission is found to be < 4000 K, implying warm CCT, whereas blue emission is > 4000 K, implying cool CCT. The optical and physical parameters were evaluated with appropriate formulae. The density and refractive index range from 3.710 gcm^− 3^ to 4.112 gcm^− 3^ and 2.356 to 2.368, respectively. The energy band gap varies from 3.154 eV to 3.110 eV. Metallization criterion and the electronic oxide polarizability vary in the range from 0.397 to 0.394 and 4.243 Å to 4.376 Å, respectively. By considering their structural, thermal, optical, and luminescence properties, the prepared glasses are promising materials for optical technologies, including LEDs and lasers.

## Introduction

Glass has evolved significantly beyond its traditional roles in basic applications, such as architecture and containment. In recent times, glass technology has witnessed remarkable advancements, notably in the domains of optical and luminescent functionalities. These advances are driven mainly by the integration of rare earth ions (REIs), transition metal oxides (TMOs), and heavy metal oxides (HMOs), which unlock doors to next-generation technologies in photonics, optoelectronics, and health care^1–6^. Among different glass systems, tellurium oxide (TeO_2_) based glasses have scientific and technological curiosity owing to their distinctive features, such as low phonon energy, lower melting temperatures, good chemical and thermal stability, minimized crystallization tendencies, and higher refractive indices^7–10^. When tellurium oxide (TeO_2_) combines with boric oxide (B_2_O_3_), which is a glass-forming compound known for its small cation size, longer bond length, and boron trivalence, borotellurite glasses are formed^11–13^. Borotellurite glasses possess advantageous features due to the combination of B_2_O_3_ and TeO_2_, including low phonon energy, enhanced refractive index, improved transparency, and suitability as an appropriate host for REE. Therefore, borotellurite glasses have gained significance due to their versatility and have been proposed to be more suitable for optoelectronics and photonic applications^1,14–18^.

HMOs such as PbO act as a network modifier in borotellurite glasses by disrupting B$\:-$O and Te$\:-$O bonds, forming non-bridging oxygens (NBOs) and creating an open structure. This modification enhances both physical and optical properties, notably increasing density and refractive index, which supports optoelectronic applications^19–23^. In combination with REIs, it boosts luminescence, benefiting solid-state lasers and photonic devices^3,21,24–31^. In addition, the incorporation of HMOs in the glass system energized by REIs has been explored, with the aim of achieving lower phonon energy, resulting in noteworthy features such as devitrification resistance, increased chemical durability, and reduced multi-phonon relaxation^30,32^. Incorporation of transition metal oxides (TMOs), such as zinc oxide (ZnO), enhances network modification due to their low crystallization tendency and wide glass-forming range. Its non-toxic, non-hygroscopic nature also makes it suitable for glass hosts^4,33–35^. During glass formation, ZnO also reduces the melting point, increases the refractive index, and serves as a stabilizer in the glass matrix. ZnO could decrease the glass transition temperature with enhanced thermal stability^9,36,37^. The alkali metal ions, such as sodium (Na), enhance the solubility of REIs, alter the glass structure by breaking bonds with network formers like B_2_O_3_ and TeO_2_, and facilitate the conversion between BO_3_ and BO_4_ units and TeO_4_ to TeO_3_ units. They also boost the chemical durability of B_2_O_3_$\:-$TeO_2_ glass system^20,32,38–40^.

Rare earth elements (REEs) such as europium, praseodymium, and holmium are vital to modern technology due to their distinctive features, contributing significantly to advancements in energy, defense, healthcare, and environmental fields^14,41–46^. Their unique intra-4f shell optical transitions enhance the optical and physical properties of inorganic glasses, making REEs-doped glasses a growing focus in research for technological innovations^35,47–50^. Among REEs, praseodymium (Pr^3+^) exhibits strong optical absorption in the UV, visible, and near-IR regions, making it suitable for pumping sources. The Pr^3+^ ions emit across the primary colors (RGB) with varying intensities, influenced by the surrounding ligand field. Their emission wavelengths and intensities are determined by 4f-4f and 4f-5d electronic transitions^32^. Their multiple energy levels enable simultaneous blue, green, and red emissions for visible lasers, along with IR emissions for optical amplification, and photovoltaic energy conversion. In addition, 1.3 μm emission, strong 1.6 μm emission has been observed in selenide glasses, supporting their use in U-band optical amplification^51–55^.

Damdee B et al.^56^ reported the influence of Pr^3+^ -ions on the optical features of lead-borate glasses, which absorb ultraviolet (UV) and near-infrared (NIR) photons through ^3^H_4_ transitions. They also reported the strongest emission in the red region, which is suitable for emitting devices. Wantana N et al.^57^ explored the Pr^3+^ doped Na-Al-Gd-phosphate glass systems. They reported that as Pr^3+^ increases, the density and refractive index increase, and absorption in the visible and NIR region. They also reported the strongest emission at the ^1^D_2_ → ^3^H_4_ (600 nm) transition with an emission colour tunable from pink to reddish-orange depending on the excitation, and suggested it for tunable light sources. Gracie P et al.^58^ have investigated the luminescence characteristics of Pr^3+^-doping multi-component silicate photonic films, synthesized by the spin-coating technique. The film exhibited strong UV absorption in the 250 nm to 340 nm region. They reported the Burstein-Moss shift in the energy band gap. PL exhibits red and NIR emissions, which support telecommunications and display devices.

Previous studies predominantly focused on doping rare earth ions with single and binary host matrices such as borate, tellurite and phosphate glass systems for exploring structural and optical properties. The multicomponent-based boro-tellurite glass system involves glass modifiers (ZnO, PbO and Na_2_O), which have received comparatively less attention despite their potential merits, such as high refractive index and good rare earth ion solubility. However, the telluro-borate glass systems, in combination with TMOs and HMOs doped with praseodymium ions, have not been explored to a greater extent for their potential applications in photonics, optics, and laser technology. The present work addresses this gap by investigating praseodymium oxide in a B_2_O_3_-TeO_2_-ZnO-PbO-Na_2_O glass host, demonstrating that the multicomponent host effectively accommodates Pr^3+^ ions and exhibits promising optical and luminescent characteristics. Therefore, motivated by the luminescence properties of Pr^3+^ ions, the present work focused on exploring the impact of Pr_6_O_11_ concentration on the structural, thermal, optical and spectroscopic features of a multicomponent glass system: B_2_O_3_-TeO_2_-ZnO-PbO-Na_2_O, with potential applications in optics and laser technology. To get better insights, the influences of essential factors, including physical and optical parameters, were evaluated and interpreted.

### Synthesis and characterization techniques

**Fig. 1:**
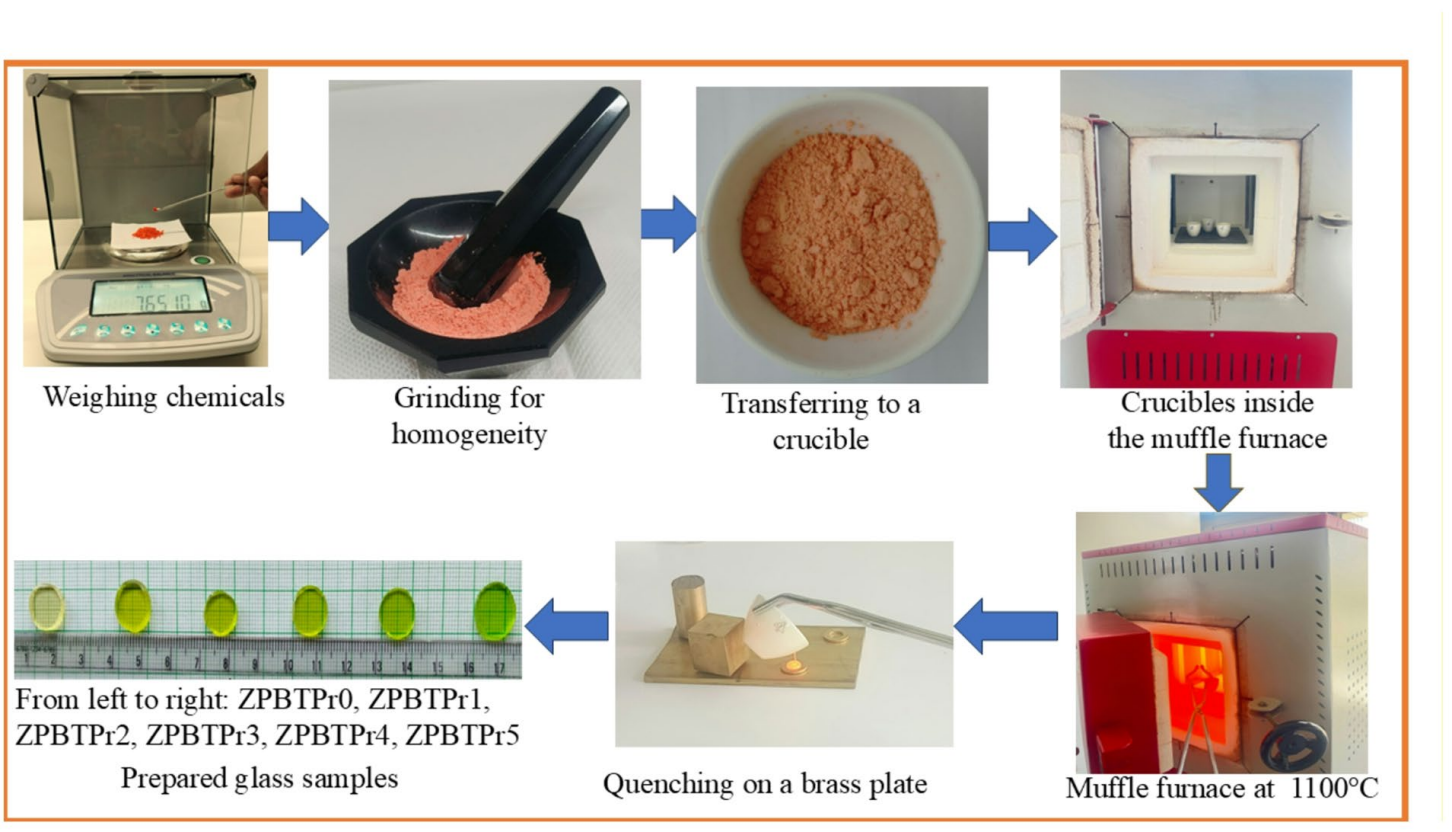
Synthesis of ZPBTPr glasses.

A novel series of Praseodymium oxide-doped zinc-lead-sodium borotellurite glasses having the composition (45-y)B_2_O_3_+20TeO_2_+20ZnO+5Pb_3_O_4_+10Na_2_O+yPr_6_O_11_, where y = 0.0, 0.3, 0.6, 1.0, 1.5, and 2.0 mol%, were prepared by the melt-quenching approach. Hereupon, the prepared glasses are coded as ZPBTPr0, ZPBTPr1, ZPBTPr2, ZPBTPr3, ZPBTPr4, and ZPBTPr5, corresponding to 0.0, 0.3, 0.6, 1.0, 1.5, and 2.0 mol% of Pr_6_O_11_, respectively. Chemicals including Tellurium Dioxide (TeO_2_) (purity-99.999%, Sisco Research lab Pvt Ltd.), Boric acid (H_3_BO_3_) (purity-99.5%, Sisco Research lab Pvt. Ltd), Zinc oxide (ZnO) (purity-99.0% Thermo Fisher scientific India Pvt. Ltd.), Lead oxide (Pb_3_O_4_) (purity-99.0%, s d fine-CHEM Ltd), Sodium carbonate (Na_2_CO_3_) (purity-99.5%, s d fine CHEM Pvt. Ltd.), and Praseodymium oxide (Pr_6_O_11_) (purity- 99.9%, Loba Chemie Pvt. Ltd.), were weighed precisely in accordance with molar ratios. The raw materials were then ground thoroughly in an agate mortar with a pestle for 30 min to ensure a homogeneous mixture and then transferred into a crucible. The crucible was placed in a muffle furnace, and the temperature was raised at a rate of 10${\rm ^\circ C}$ per minute until 1100 ${\rm ^\circ C}$ under normal atmospheric conditions. This temperature was maintained for about 15 min. Then the melt was gently stirred for homogeneity and then kept in the furnace for about 30 min. The molten glass was subsequently poured into brass rings placed on a brass plate at room temperature and quickly pressed by a brass weight to suppress crystallization. A systematic illustration of the synthesis of glasses is shown in Fig. 1.

The synthesized glass samples were obtained in both powdered and bulk forms, which are appropriate for characterization. The amorphous state of synthesized glasses was confirmed through a Bruker-D8 Focus X-ray diffractometer (XRD), which recorded X-ray diffraction patterns from 10$\:^\circ\:$ − 80$\:^\circ\:$. Structural modifications and the presence of functional groups were examined through an attenuated total reflectance-Fourier transform spectrometer (ATR-FTIR) by recording spectra in the range of 400 cm^− 1^ – 1600 cm^− 1^, and a Raman microscope equipped with a pro-Raman system, recording spectra between 350 cm^− 1^ and 2000 cm^− 1^. EVO 10 scanning electron microscope, integrated with an energy-dispersive X-ray spectroscopy (SEM-EDX) and featuring a tungsten filament, was utilised for surface morphology and elemental analysis. The SEM images and elemental data were obtained with an acceleration voltage of 0.2 kV to 10 kV. Using an Ultraviolet (UV)-visible spectrometer (Model UV-1900I), the UV-visible spectra were recorded in the range of 300 nm to 800 nm. Thermal analysis was performed using differential scanning calorimetry (DSC) measurements with a Hitachi Nexta STA 200 model, within a temperature range of 30 ${\rm ^\circ C}$ to 585 ℃ at a constant heating rate of 10 ℃/min. The analysis was performed on all the ZPBTPr powdered samples, which were placed in an alumina crucible, with sample masses ranging between 5 mg and 12 mg. The uncertainty in measuring the glass transition temperature and crystallization temperature is within $\:\pm\:1{\rm ^\circ C}$. A spectrofluorometer (Model-Horiba Jobin Yvon-FluoroMax4) coupled with a 150 W ozone-free xenon arc lamp was employed to explore luminescence features. The spectra were recorded in the visible region, from 460 to 720 nm at an excitation wavelength of 444 nm. Density of glasses determined via Archimedes’ principle. The vital optical and physical parameters are determined by suitable formulae. A systematic assessment of structural, physical, optical, thermal and luminescence features provides valuable insights into the suitability of synthesized glasses for optical and laser applications.

## Results and discussions

### X-ray diffraction (XRD) studies

**Fig. 2:**
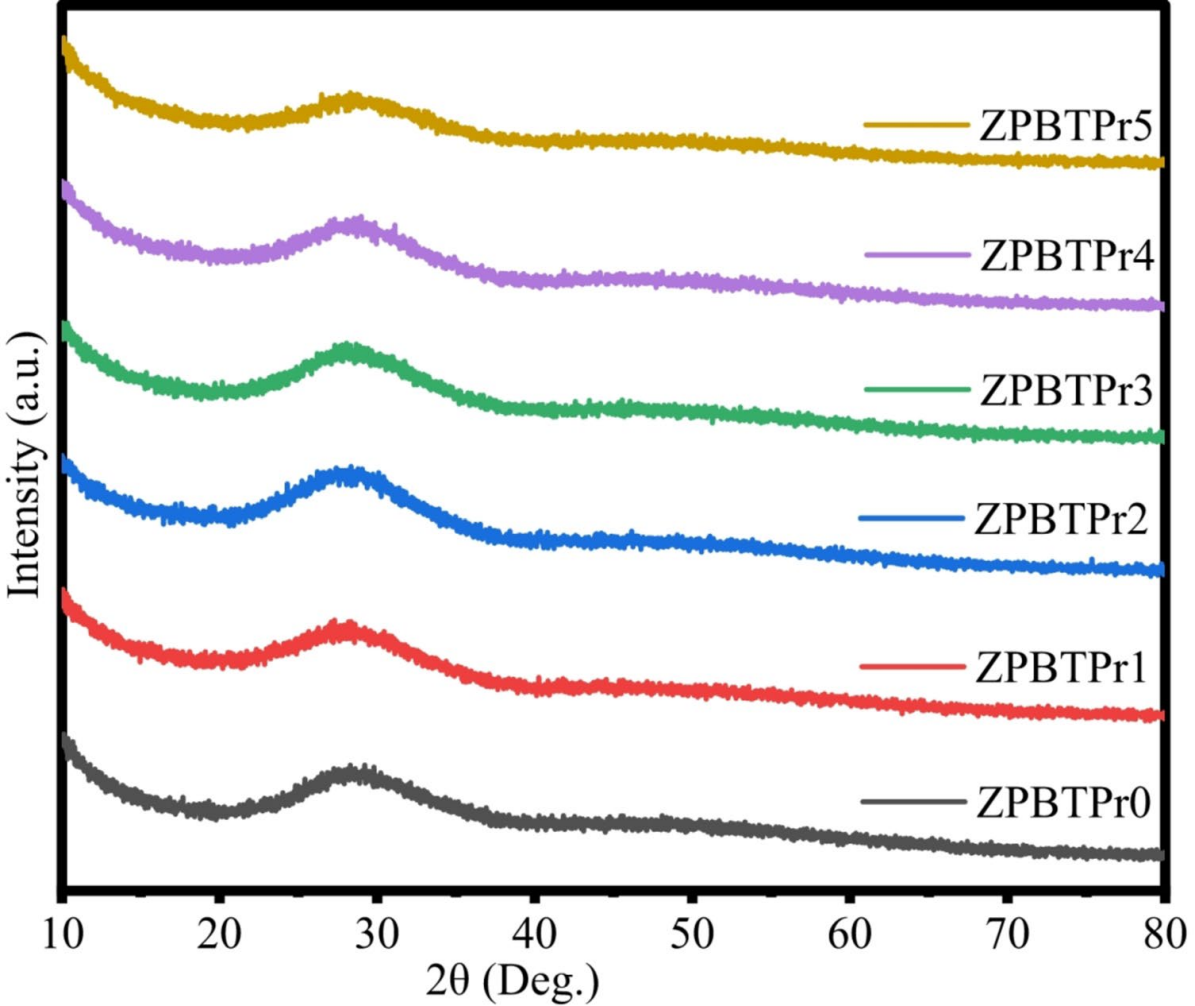
XRD patterns of ZPBTPr glasses.

The non-crystalline state of all the synthesized undoped and praseodymium-doped glasses was assessed through the XRD technique. The XRD pattern obtained in the range of 2$\:\theta\:=$ 10$\:^\circ\:$ − 80$\:^\circ\:$ and depicted in Fig. 2. The noticeable broad hump exhibits between 20$\:^\circ\:$ − 40$\:^\circ\:$ and no sharp peak, indicating the absence of long-range periodic arrangement of atoms in ZPBTPr glasses, in the entire range, provides evidence for the glassy nature of ZPBTPr samples. It has been noted that even at a higher concentration of praseodymium, the sample retains the non-crystalline state^21,59^.

### Thermal properties

**Fig. 3:**
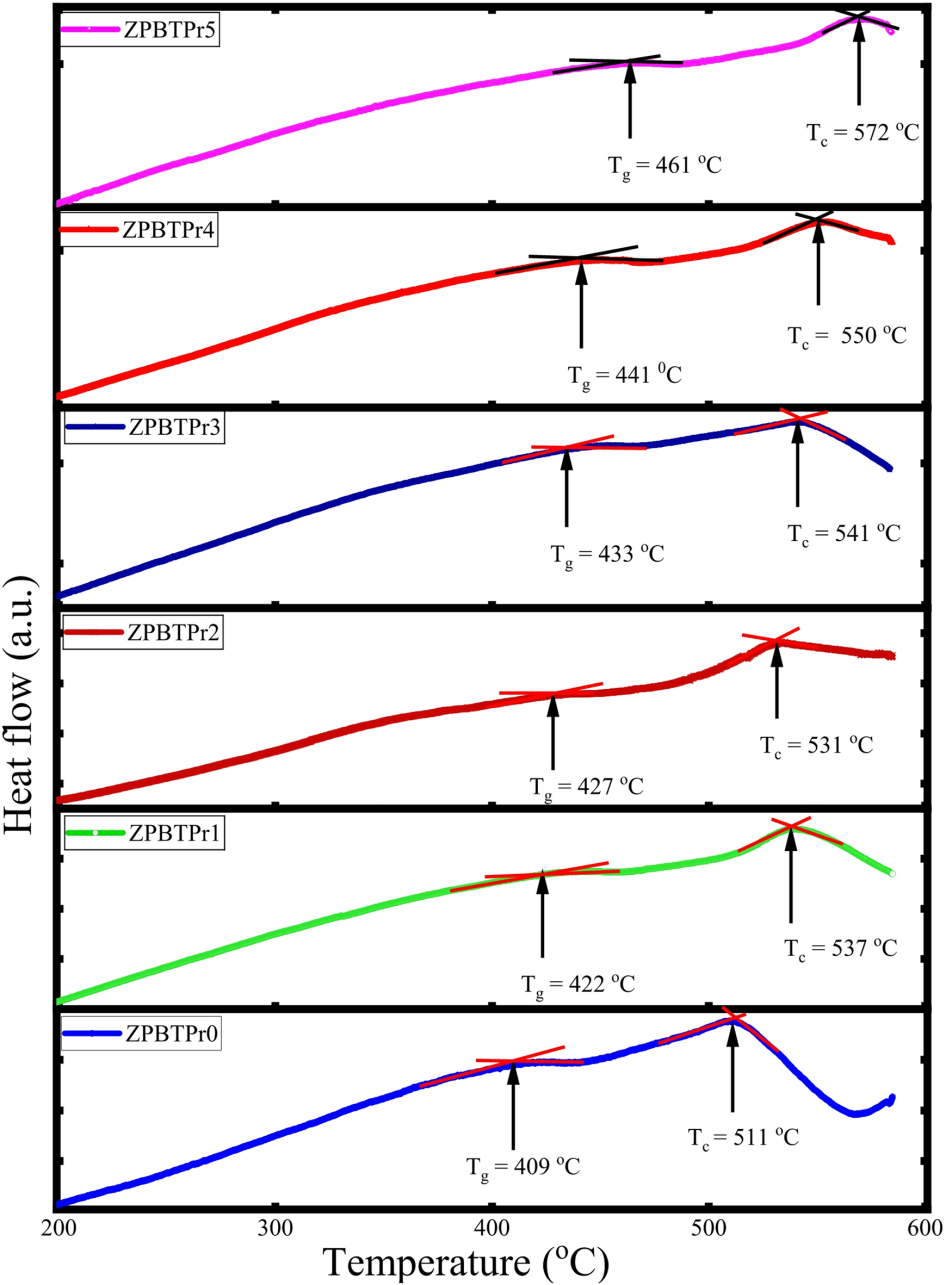
DSC patterns of ZPBTPr glasses.

The thermal analysis of ZPBTPr glasses through differential scanning calorimetry (DSC) is presented in Fig. 3. The DSC pattern exhibits an endothermic process at the glass transition and an exothermic process at crystallization. In the DSC pattern, the starting point of the glass transition range was considered for T_g_. The observed glass transition temperatures ($\:{T}_{g}$) and crystallization temperatures ($\:{T}_{c}$) are tabulated in Table 1. $\:{T}_{g}$ values vary from 409 ${\rm ^\circ C}$ to 461 ${\rm ^\circ C}$, whereas T_c_ values vary from 511 ${\rm ^\circ C}$ to 572 ${\rm ^\circ C}$. DSC thermal analysis reveals that as Pr_6_O_11_ increases, $\:{T}_{g}$ increases and enhances thermal stability and resistance to devitrification, which can be attributed to variations in physical parameters, such as density and ionic radius, as discussed in Sect. 3.2, as well as structural modifications resulting from the conversion of TeO_4_ to TeO_3+1_ or TeO_3_. An increase in $\:{T}_{c}$ implies enhanced resistance to nucleation, indicating higher thermal energy required for nucleation. Thermal stability ($\:\varDelta\:T$) is a vital factor that reflects the degree of disorder in non-crystalline materials. $\:\varDelta\:T$ is calculated by using a relation $\:{{\Delta\:}\mathrm{T}=T}_{c}-{T}_{g}$ and is found to vary from 102 ${\rm ^\circ C}$ to 111 ${\rm ^\circ C}$. Glasses with $\:\varDelta\:T$ values above 100 ${\rm ^\circ C}$ are typically regarded as thermally stable^60^. Hence, ZPBTPr glasses are thermally stable and variation in $\:\varDelta\:T$ is associated with changes in the BOs within the glass network.

**Table 1:** Thermal properties of ZPBTPr glasses: Glass transition temperature ($\:{T}_{g}$), Crystallization temperature ($\:{T}_{c}$), Thermal stability ($\:\varDelta\:T$).

Sample code	ZPBTPr0	ZPBTPr1	ZPBTPr2	ZPBTPr3	ZPBTPr4	ZPBTPr5
$\:{T}_{g}\:(\pm\:1\:{\rm ^\circ C}$)	409	422	427	433	441	461
$\:{T}_{c}\:(\pm\:1\:{\rm ^\circ C}$)	511	537	531	541	550	572
$\:\varDelta\:T\:(\pm\:\:1\:{\rm ^\circ C}$)	102	115	104	108	109	111

## Physical properties

### Molar volume ($\:{\boldsymbol{V}}_{\boldsymbol{m}}$), density ($\:\boldsymbol{\rho\:}$), boron-boron distance ($\:{\boldsymbol{d}}_{\boldsymbol{B}-\boldsymbol{B}}$), and oxygen packing density (OPD)

The physical parameters of ZPBTPr glasses, such as ρ, V_m_, d_B−B_, and OPD, are evaluated by the following relations^61^,

1 $$\:\rho\:=\left(\frac{{W}_{g\mathrm{a}}}{{W}_{ga}-{W}_{gt}}\right){W}_{t}$$

2 $$\:{V}_{m}={M}_{\mathrm{a}v}/\rho\:$$

3 $$\:{d}_{B-B}={\left[\frac{{V}_{m}^{B}}{{N}_{A}}\right]}^{1/3}$$

4 $$\:OPD\:=\:(\rho\:\times\:a\times\:{10}^{3})/{M}_{\mathrm{a}v}$$

Where,

$\:{W}_{g\mathrm{t}}\:and\:{W}_{ga}$ represent the weight of glass in air and toluene, $\:{\rho\:}_{t}=$ 0.8635 gcm^−3^, Density of toluene, $\:{M}_{\mathrm{a}v}=$ Average molecular weight, $\:a=$ Number of Oxygen atoms/formula unit, $\:{N}_{A}=6.0223\times\:{10}^{23}\mathrm{g}/\mathrm{m}\mathrm{o}\mathrm{l}$, Avogadro’s number, $\:{V}_{m}^{B}=\frac{{V}_{m}}{1-{X}_{B}}$, is the volume corresponding to 1 mol of B-atoms, $\:{X}_{B}=$ Molar fraction of B_2_O_3_.

**Fig. 4:**
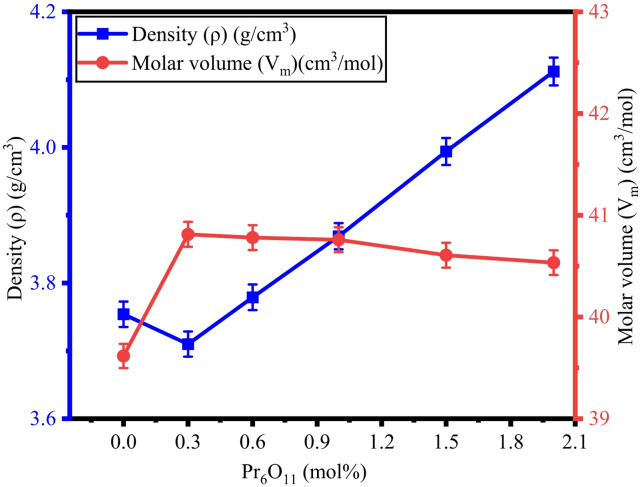
Variation of ρ vs. V_m_ of ZPBTPr glasses.

**Fig. 5:**
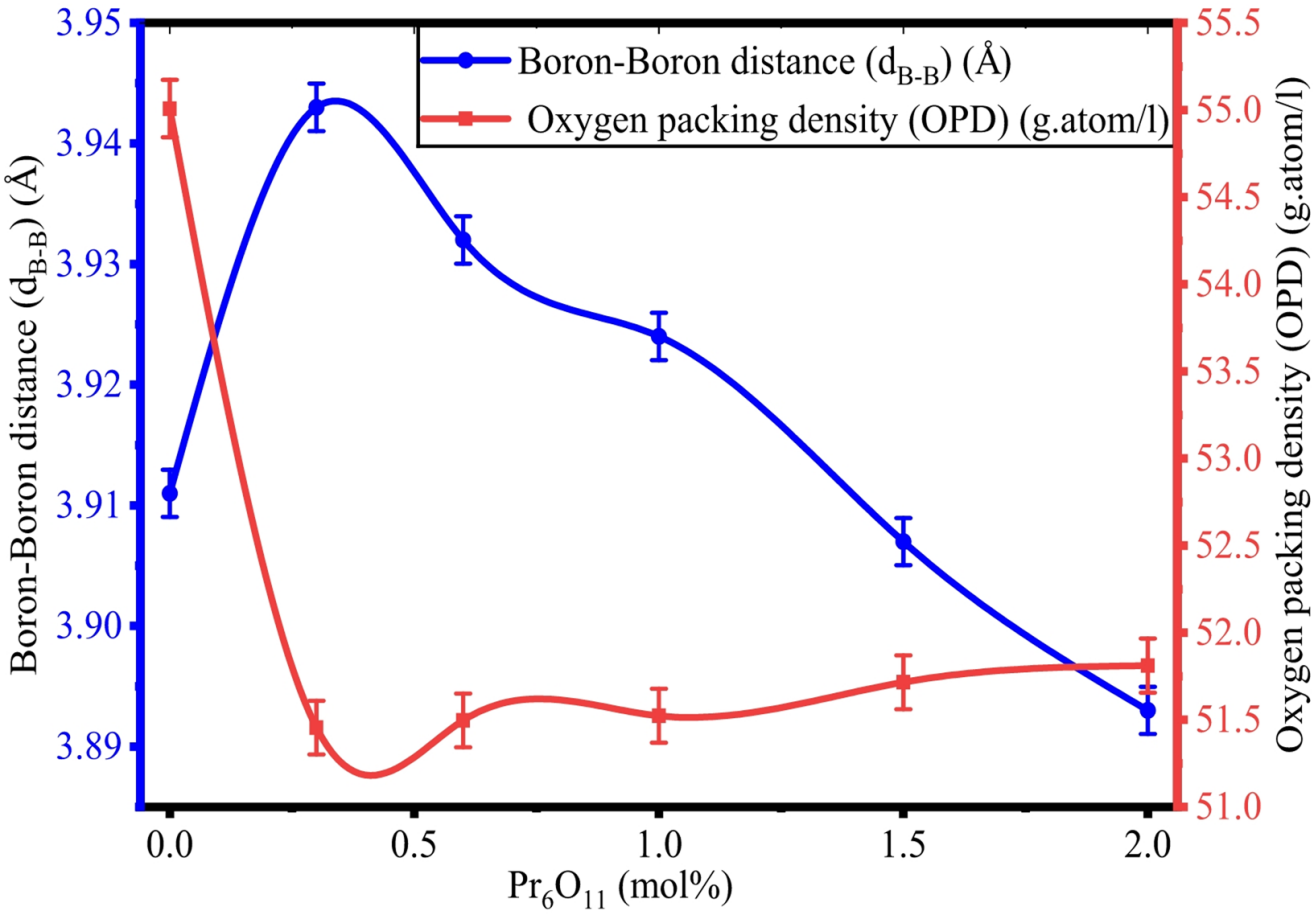
Variation of d_B−B_ vs. OPD in ZPBTPr glasses.

By utilizing Archimedes’ principle, the density (ρ) of ZPBTPr glasses was determined by measuring the weights in air and toluene. The determined values of ρ, V_m_, d_B−B_, and OPD are tabulated in Table 2. The ρ and V_m_ vary from 3.710 g/cm^3^ to 4.112 g/cm^3^ and 39.616 cm^3^/mol to 40.812 cm^3^/mol. The values were found to show an opposite trend, as shown in Fig. 4. However, there is a small change in the values of V_m_, and this is attributed to the replacement of B-atoms by Pr^3+^ ions, which have a high molecular weight and structural modification influenced by the presence of ZnO and PbO, leading to more compact local regions rather than expanding^47,62^. The maximum density was noted at a high concentration of Pr^3+^ ions (x = 2.0 mol%). OPD and d_B−B_ values range from 55.008 g.atom/l to 51.811 g.atom/l and from 3.943 Å to 3.893 Å. The variation of OPD and d_B−B_ is shown in Fig. 5. The variations observed in OPD and d_B−B_ are in good correlation with the molar volume and density. As the Pr^3+^ content increases, the interaction of TeO_2_, B_2_O_3_, ZnO, PbO, and Na_2_O takes place and alters the network by rearranging the B-atoms and the formation of bridging oxygens (BOs)^62^.

### Polaron radius ($\:{\boldsymbol{r}}_{\boldsymbol{p}}$), interionic distance ($\:{\boldsymbol{r}}_{\boldsymbol{i}}$), and field strength ($\:\boldsymbol{F}$)

The parameters such as $\:{r}_{i}$, $\:{r}_{p}$, and $\:F$ for ZPBTPr glasses are determined through the relations as follows^63^,

5 $$\:{r}_{i}=\:{\left[\frac{1}{{N}_{i}}\right]}^{1/3}$$

6 $$\:{r}_{p}=0.5\:{\left[\frac{\pi\:}{{6N}_{i}}\right]}^{\raisebox{1ex}{$1$}\!\left/\:\!\raisebox{-1ex}{$3$}\right.}$$

7 $$\:F=\:\frac{Z}{{r}_{p}^{2}}$$

Where,

$\:{N}_{i}=\frac{x\rho\:{N}_{A}}{{M}_{\mathrm{a}v}}$, is the concentration of ions, $\:x=$ mol% of dopant, $\:Z=$ Valency of cation.

**Fig. 6:**
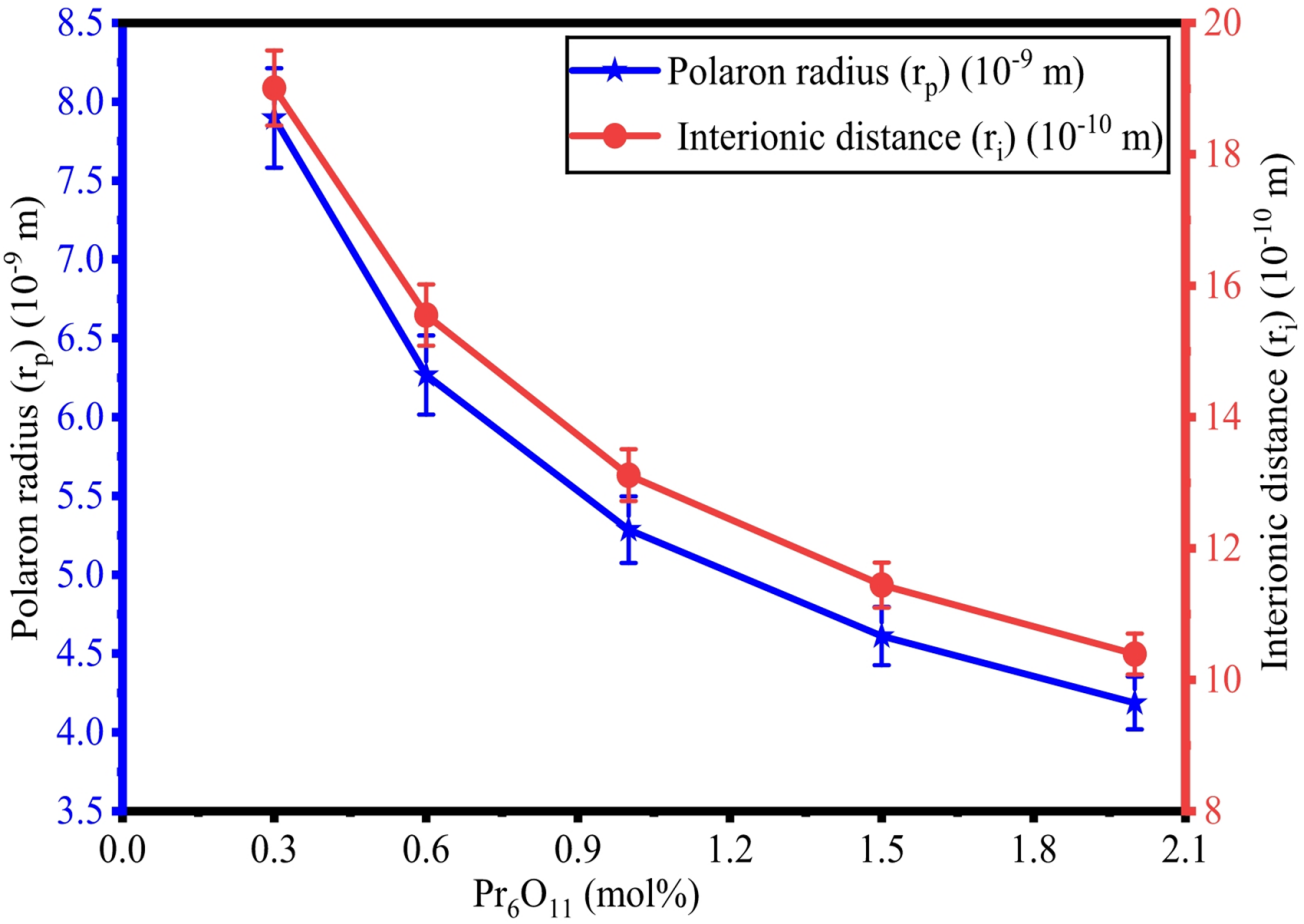
Variation of $\:{r}_{p}$ vs. $\:{r}_{i}$ in ZPBTPr glasses.

**Fig. 7:**
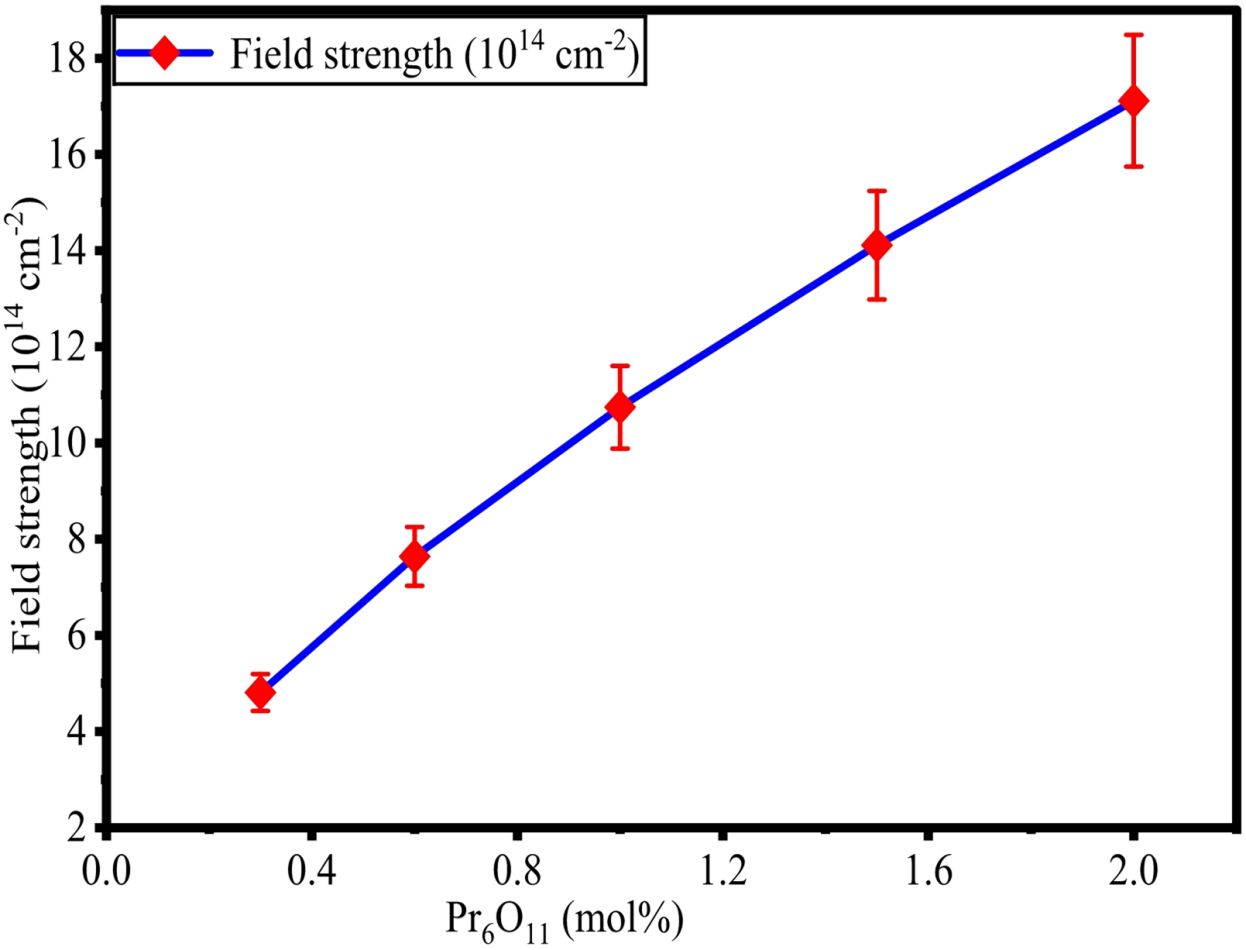
Variation of $\:\mathrm{F}$ vs. Pr_6_O_11_ in ZPBTPr glasses.

The determined values of $\:{r}_{i}$, $\:{r}_{p}$, and $\:F$ for ZPBTPr glasses are tabulated in Table 2 and their variations depicted in Figs. 6 and 7. The $\:{r}_{i}$, and $\:{r}_{p}$ values vary from 1.901 nm to 1.039 nm and 7.897 Å to 4.186 Å. The changes observed in the $\:{r}_{i}$, $\:{r}_{p}$, and $\:F$ values can be linked to the increasing concentration of Pr^3+^ ions. As Pr^3+^ ions increase, they occupy the interstitial positions in the glass network, and modify the network producing BOs, resulting in the compact structure. Hence, both polaron radius and interionic distance show a decreasing tendency with an increase in Pr^3+^ ions^42,62,64^. The electric field exerted by the ions is evaluated through the variation in field strength (F) with an increase in Pr^3+^ ions. The F values were found to increase with an increase in Pr^3+^ content. This is due to the positive charge of Pr^3+^ ions and a larger ionic radius. The incorporation of Pr^3+^ ions into the glass network alters the structure by forming BOs and increasing the electric field around the ions^42,62,64^. As a result, the decreasing trend in $\:{r}_{i}$, and $\:{r}_{p}$, and an increasing trend in $\:F$ are linked to structural modification in ZPBTPr glasses with increasing Pr^3+^ ion concentration.

**Table 2:** Physical parameters of ZPBTPr glasses: Molar volume (V_m_), Density (ρ), Ion concentration (N_i_), Boron-Boron distance (d_B−B_), Oxygen packing density (OPD), Interionic radius (r_i_), Polaron radius (r_p_), and Field strength (F).

Sample code	ZPBTPr0	ZPBTPr1	ZPBTPr2	ZPBTPR3	ZPBTPr4	ZPBTPr5
	$\:\pm\:0.001$	$\:\pm\:0.001$	$\:\pm\:0.001$	$\:\pm\:0.001$	$\:\pm\:0.001$	$\:\pm\:0.001$
ρ (g/cm^3^)	3.754	3.710	3.779	3.869	3.994	4.112
V_m_ (cm^3^/mol)	39.616	40.812	40.780	40.759	40.607	40.534
N_i,_ 10^20^ (ions/cm^3^)	-	1.327	2.658	4.432	6.673	8.914
d_B−B_ (Å)	3.911	3.943	3.932	3.924	3.907	3.893
OPD (g.atom/l)	55.008	51.455	51.496	51.523	51.715	51.811
r_i_ (nm)	-	1.901	1.555	1.311	1.144	1.039
r_p_ (Å)	-	7.897	6.266	5.286	4.611	4.186
F, 10^14^ (cm^− 2^)	-	4.809	7.639	10.742	14.112	17.115

## Structural studies

### ATR-Fourier transform infrared (ATR-FTIR) spectroscopy

**Fig. 8:**
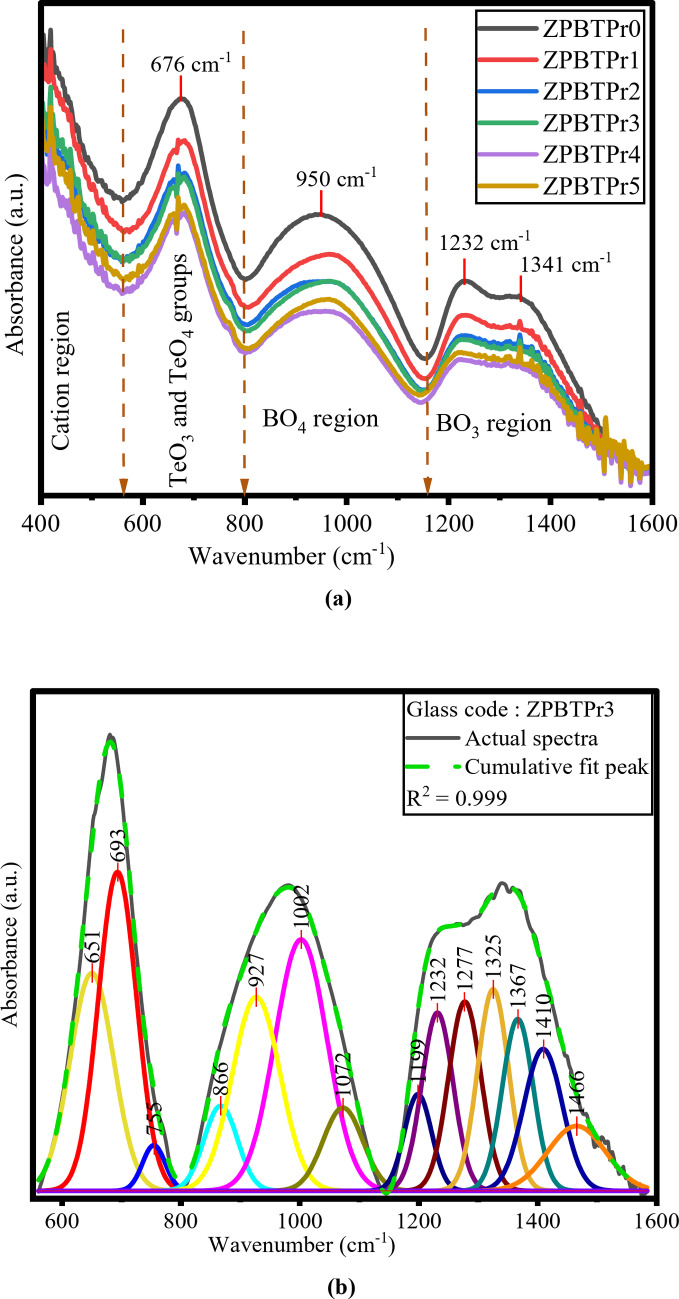
**(a)** ATR-FTIR of ZPBTPr glasses. **(b)** Deconvolution of the ATR-FTIR of ZPBTPr3 glass.

ATR-FTIR is an effective and non-destructive method applied in structural investigations. This approach was used to examine the spectral peaks, which correspond to the vibration modes of distinct functional groups within the glass matrix. The absorption regions between 800$\:-$1200 cm^− 1^ and 1200$\:-$1600 cm^− 1^ in boro-tellurite glasses are typically attributed to the stretching and bending vibrations of BO_4_ and BO_3_ units^65^. The range 600$\:-$800 cm^− 1^ is associated with stretching vibrations of TeO_3_ and TeO_4_ units of tellurite structure. Figure 8**(a)** illustrates the FTIR spectra of ZPBTPr glasses, recorded in the range of 400–1600 cm^− 1^. According to prior studies on tellurite and borate glasses, the bands appearing at 950 cm^− 1^, 1232 cm^− 1^, and 1431 cm^− 1^ in ZPBTPr glasses spectra correspond to BO_4_ and BO_3_, respectively. The absorption band near 676 cm^− 1^ is attributed to the stretching vibrations of TeO_4_ and TeO_3_ configurations associated with BOs. The presence of network modifiers such as ZnO, PbO, and Na_2_O in the composition changes TeO_4_ to TeO_3_ or TeO_3+1_ units. The absorption region $\:<$560 cm^−1^ can be attributed to stretching vibrations of Pb – O and Zn – O bonds^38,66^.

The spectra reveal that an increase in Pr^3+^ ions leads to variations in band intensity and noticeable shifts in position. These modifications are associated with the B – O stretching vibrations of BO_3_ structural groups in various borate configurations, such as ortho, pyro, and metaborate groups. Deconvolution of all ZPBTPr spectra was performed using the peak fit tool in Gaussian functions to obtain the detailed information for functional groups from overlapping peaks caused by closely spaced vibrational modes. Figure 8**(b)** shows a typical deconvoluted spectrum (ZPBTPr3). Band assignments for ZPBTPr glasses are summarized in Table 3.

**Table 3:** ATIR-FTIR band assignments of ZPBTPr glasses.

Functional units	Wavenumber (cm^− 1^)	Assignments	Ref.
TeO_4_	631–698	Te – O bond vibrations in TeO_4_. Stretching vibrations Te – O linkages.	^37,67,68^
TeO_3_	703–805	TeO_3_ and TeO_3+1_ polyhedra involving vibrations of Te – O and Te – O – Te bonds. Asymmetric stretching vibrations of Te = O bonds in TeO_3_.	^61,67,69^
BO_4_	866–907	Tri-, tetra-, and penta-borate groups with B – O stretching in BO_4_.	^37,67^
	908–1193	B – O Stretching in BO_4_ from di-borate units.	^67^
BO_3_	1206–1367	Bending and stretching vibrations of B-O-B in BO_3_ units. Vibrations of B – O in meta, pyro, and ortho-borate groups.	^37,67^
	1377–1478	BO_3_ units have asymmetric stretching vibrations.	^37^

### Raman studies

**Fig. 9:**
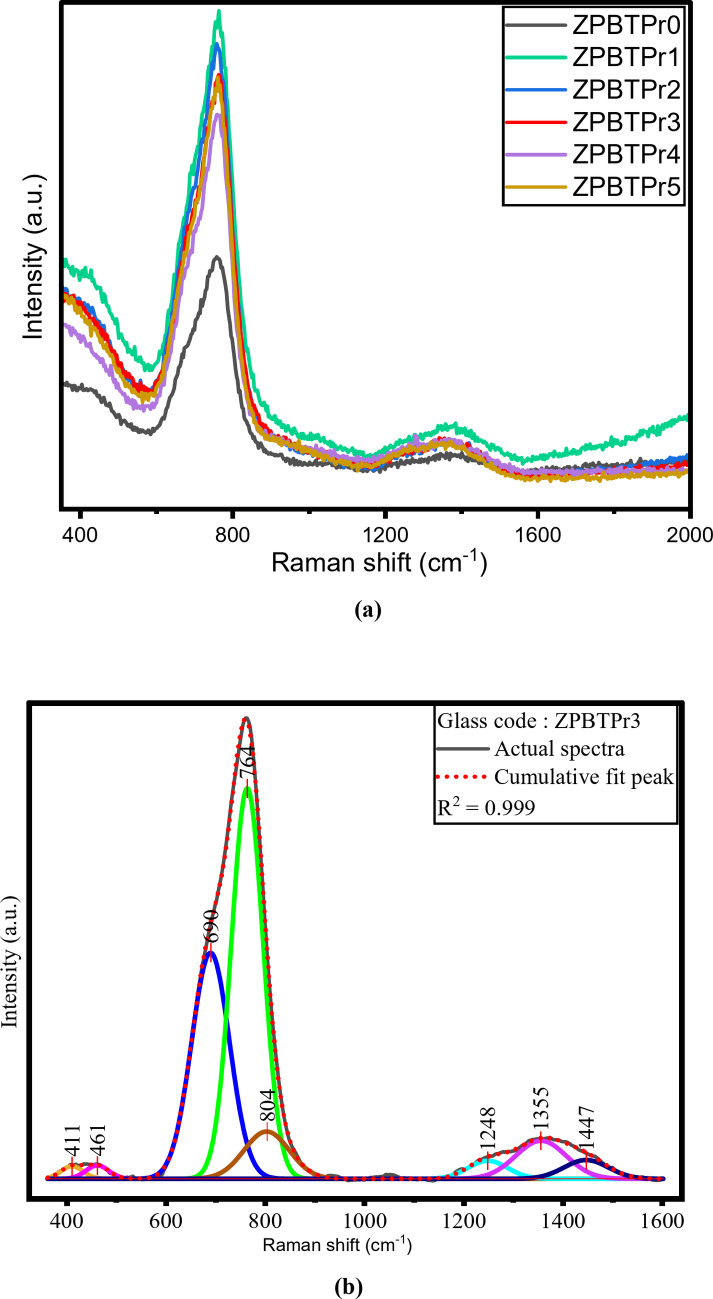
**(a)** Raman spectra of ZPBTPr glasses. **(b)** Deconvolution of the Raman spectrum of ZPBTPr3 glass.

Raman spectroscopy is an effective tool for investigating the structural and compositional properties of glass systems, making it invaluable in the design of materials for optical and photonic applications. Figure 9**(a)** shows the Raman spectra of ZPBTPr glasses, which were recorded in the range of 350–2000 cm^− 1^. A typical deconvoluted spectrum of ZPBTPr glasses (ZPBTPr3) is shown in Fig. 9**(b)**. These measurements reveal detailed information about the glass network and show the structural modifications that occurred in the ZPBTPr glasses. In the boro-tellurite glass network, boron trioxide (B_2_O_3_) primarily introduces borate groups, such as BO_3_ and BO_4_. Vibrational bands associated with BO_4_ and BO_3_ are typically found in the range of 800$\:-$1600 cm^− 1^^70–72^. Tellurite structures such as TeO_4_, TeO_3_ and TeO_3+δ,_ which exhibit stretching, bending and antisymmetric vibrations, are assigned in the region of 673–873 cm^− 1^^73,74^. Raman spectra exhibit variation in intensity with the addition of Pr^3+^ ions because of a relatively larger ionic radius and strong field strength, which induces local structural distortions in the glass network. Initially, when Pr^3+^ ions are introduced, they modify the structure by altering the speciation of Te – O and B – O, as well as the conversion of BO_3_ and BO_4_ and also TeO_4_ and TeO_3_, with changes in the NBOs and BOs. As the Pr_3+_ ions concentration increases, there is a variation in the intensity and a slight shift in the band, indicating structural rearrangement with the formation of TeO_3+1_ and TeO_3_. These structural alterations impact the bond polarizability and increase the Raman cross-sections of vibrational modes. High intensity occurs at ~ 764 cm^− 1^, due to vibration of Te – O tp units from TeO_3_ and TeO_3+1_ groups with symmetric stretching vibrations of 6-membered rings, when BO_4_ tetrahedra substitute the BO_3_ triangle. In addition to this, there is a contribution from B – O – B vibrations that occur from BO_4_ units. The peak at ~ 1325 cm^− 1^ is attributed to various borate groups such as meta, pyro, and ortho borate groups. At higher concentrations, clustering of ions and absorption of photons result in a slight decline in the intensity. In addition, local coordination with cations such as Pb^2+^ and Zn^2+^ alters the bond strengths and polarizability of neighboring bonds, resulting in a slight shift in intensity among overlapping components. In Raman spectroscopy, the highest vibrational energy corresponds to the phonon energy of the glass host matrix. In this study, the maximum phonon energy recorded is ~ 764 cm^− 1^, which is comparatively lesser than other glass systems, such as silicate, 889 cm^− 1^^75^. (However, as reported^76–78^ the pure SiO_2_ based glasses phonon energy is in the range 900 cm^− 1^ – 1200 cm^− 1^) and for phosphate glasses, 1120 cm^− 1^^75^. Raman spectra band assignments for ZPBTPr glasses are presented in Table 4.

**Table 4:** Band assignments of Raman spectra of ZPBTPr glasses.

Wavenumber (cm^− 1^)	Assignments	Ref.
~ 411	Vibrations of Zn – O and Pb – O bonds.	^79,80^
~ 461	Attributed to bending and stretching vibrations of Te$\:-$O$\:-$Te from TeO_4_, TeO_3_, and TeO_3+1_ groups.	^73,74^
670$\:-$690	Antisymmetric vibrations in TeO_3_, TeO_4_, and, TeO_3+1_ units.	^73,74^
763$\:-873$	Vibrations of Te – O tp units from TeO_3_ and TeO_3+1_ groups with symmetric stretching vibrations of 6-membered rings, where the BO_3_ triangle is substituted by BO_4_ tetrahedra. Bending vibrations of B – O – B in BO_4_ units.	^70,71^–^79^^72,81^
~ 1231	B – O asymmetric stretching vibrations in BO_4_ units.	^79^
~ 1325	Stretching vibrations in meta-, pyro-, and ortho borate units.	^82^
1421$\:-$1470	BO_2_$\:{\mathrm{O}}^{-}$ triangles linked to BO_3_ units.	^72,80^

### Scanning electron microscope with energy dispersive X-ray spectroscopy

**Fig. 10:**
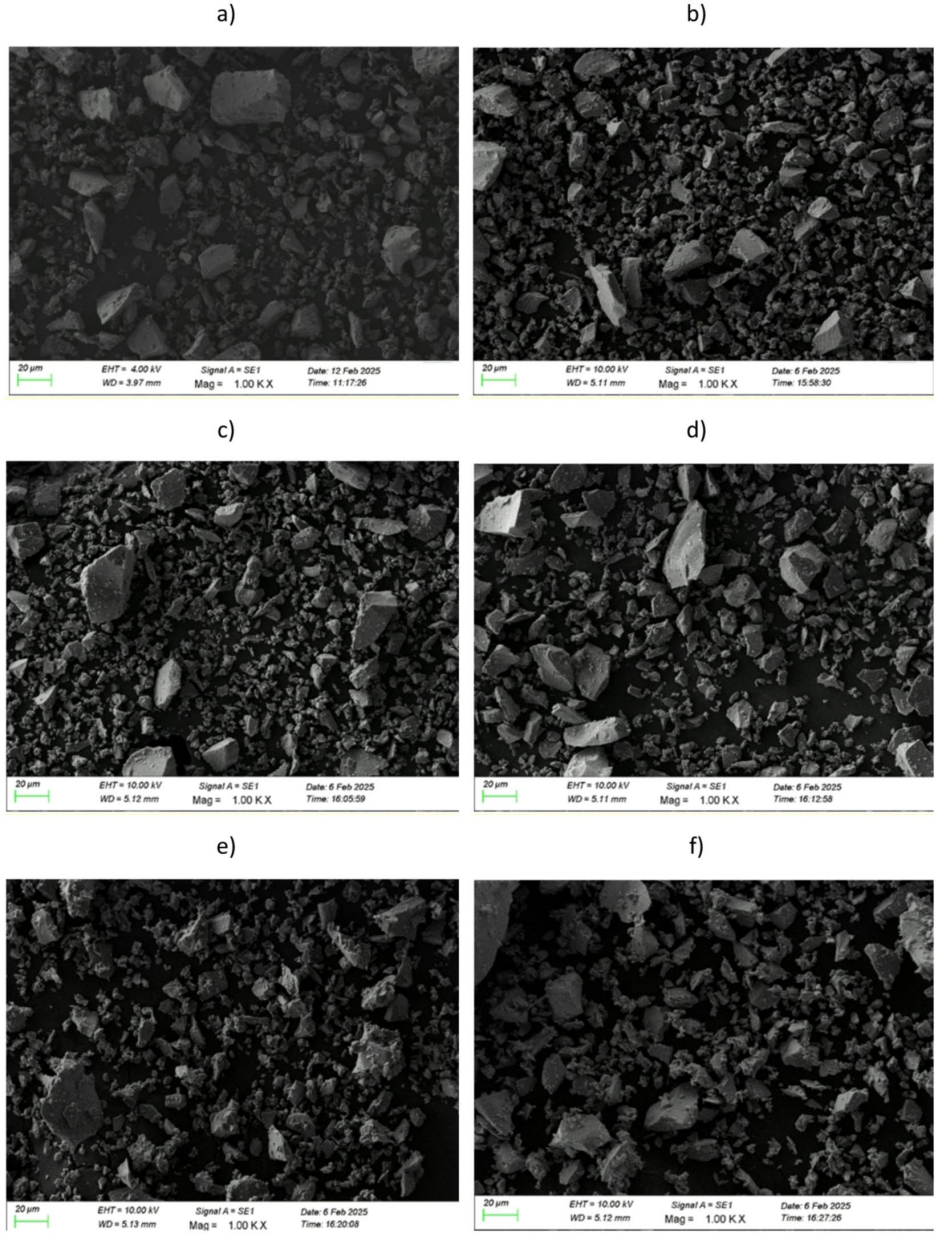
SEM micrographs of ZPBTPr glasses.

**Fig. 11:**
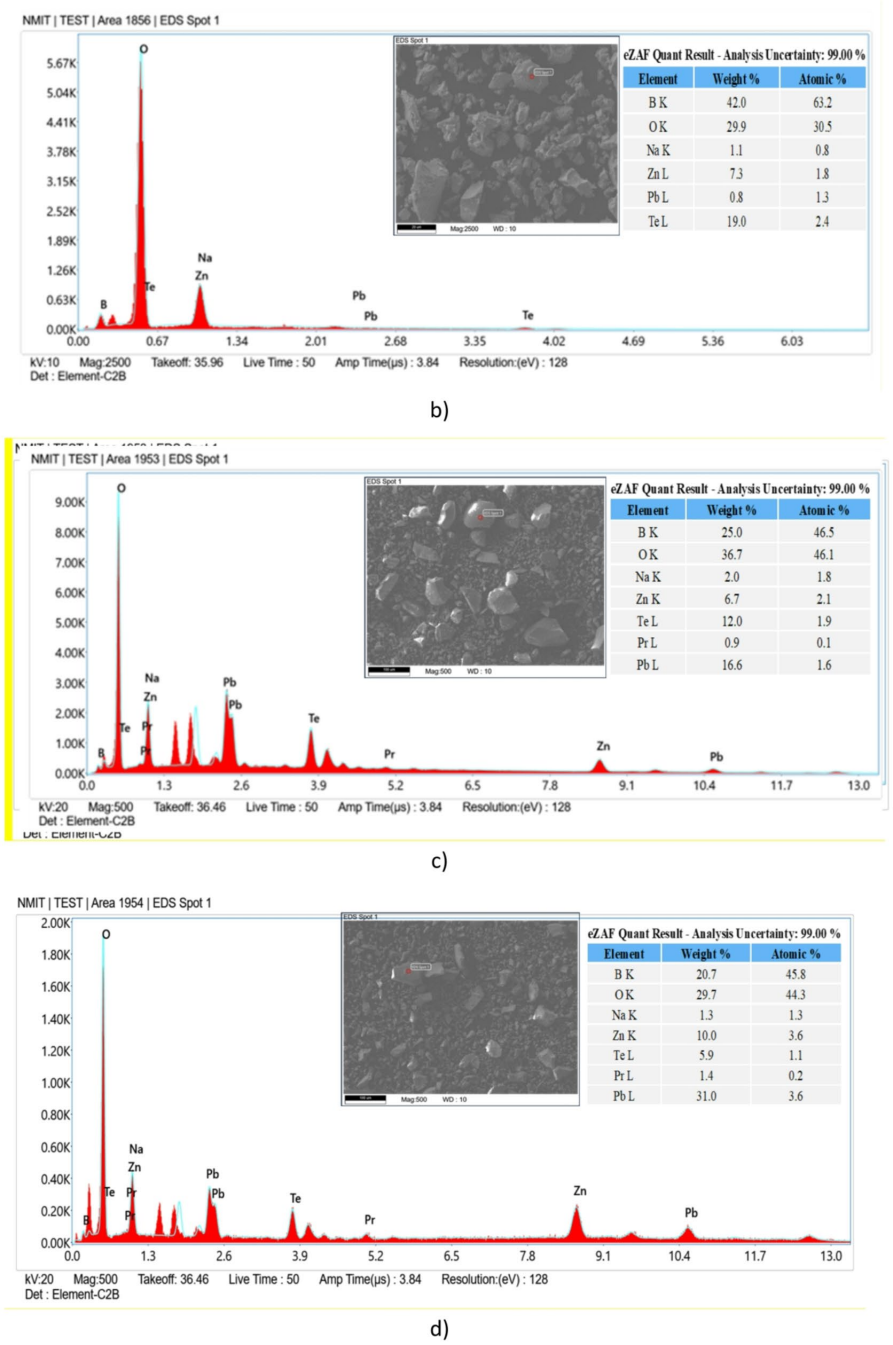

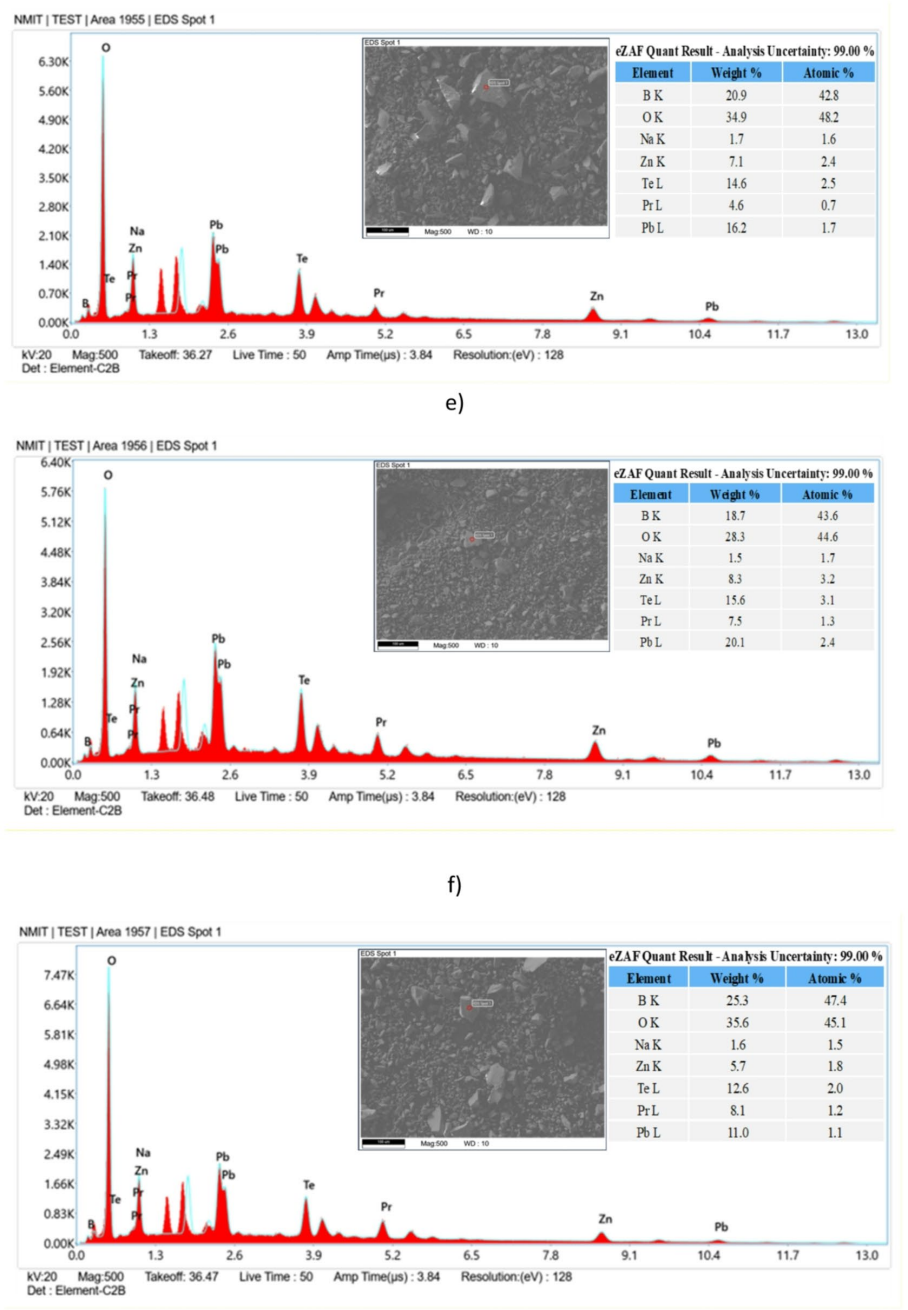
EDX spectra of ZPBTPr glasses. **a**) ZPBTPr0 **b**) ZPBTPr1 **c**) ZPBTPr2 **d**) ZPBTPr3 **e**) ZPBTPr4 **f**) ZPBTPr5.

The morphology and elemental composition of ZPBTPr glasses were analysed using a scanning electron microscope (SEM) coupled with an energy – dispersive X-ray microscope (EDX). SEM images of all ZPBTPr glasses are presented in Fig. 10, and their corresponding EDX spectra are depicted in Fig. 11. The ZPBTPr glass samples’ images reveal a relatively uniform surface^38^. However, a slight irregularity was observed in the microstructure, which can be attributed to the influence of network modifiers, including ZnO, PbO, and TeO_2_, within the glass matrix^83,84^. When Pr^3+^ ions were incorporated, and as the concentration increased, the images displayed fine granular regions over the microscale. The variations in microstructural changes are primarily due to disruptions in the tellurite and borate chains, and the subsequent generation of BOs and NBOs. The EDX analysis validated the elemental composition of ZPBTPr glasses, identifying tellurium (Te), boron (B), zinc (Zn), sodium (Na), oxygen (O), lead (Pb) and praseodymium (Pr). ZPBTPr0 glass showed distinct peaks corresponding to Te, B, Zn, Pb, O, and Na, while the absence of a praseodymium peak verified that this composition is free of praseodymium. By contrast, the spectra of Pr^3+^ ion-doped glasses exhibited a peak associated with praseodymium, along with other elemental peaks. With increasing Pr^3+^ ions, the intensity of the praseodymium became progressively more pronounced^42^. The noticeable variations in the relative intensities of elements with increasing concentration of Pr^3+^ ions are due to structural modifications. The weight% and atomic% of each element, corresponding to the EDX-spot inserted in the EDX-spectra. The data obtained from EDX shows that weight% and atomic% values do not exhibit a regular distribution since the region is not uniform, and values correspond to a particular spot or region. No impurity or extraneous elements were detected, indicating the synthesised glasses exhibit high compositional purity and maintain the intended stoichiometry.

## Optical properties

### UV-Visible absorption spectroscopy

**Fig. 12:**
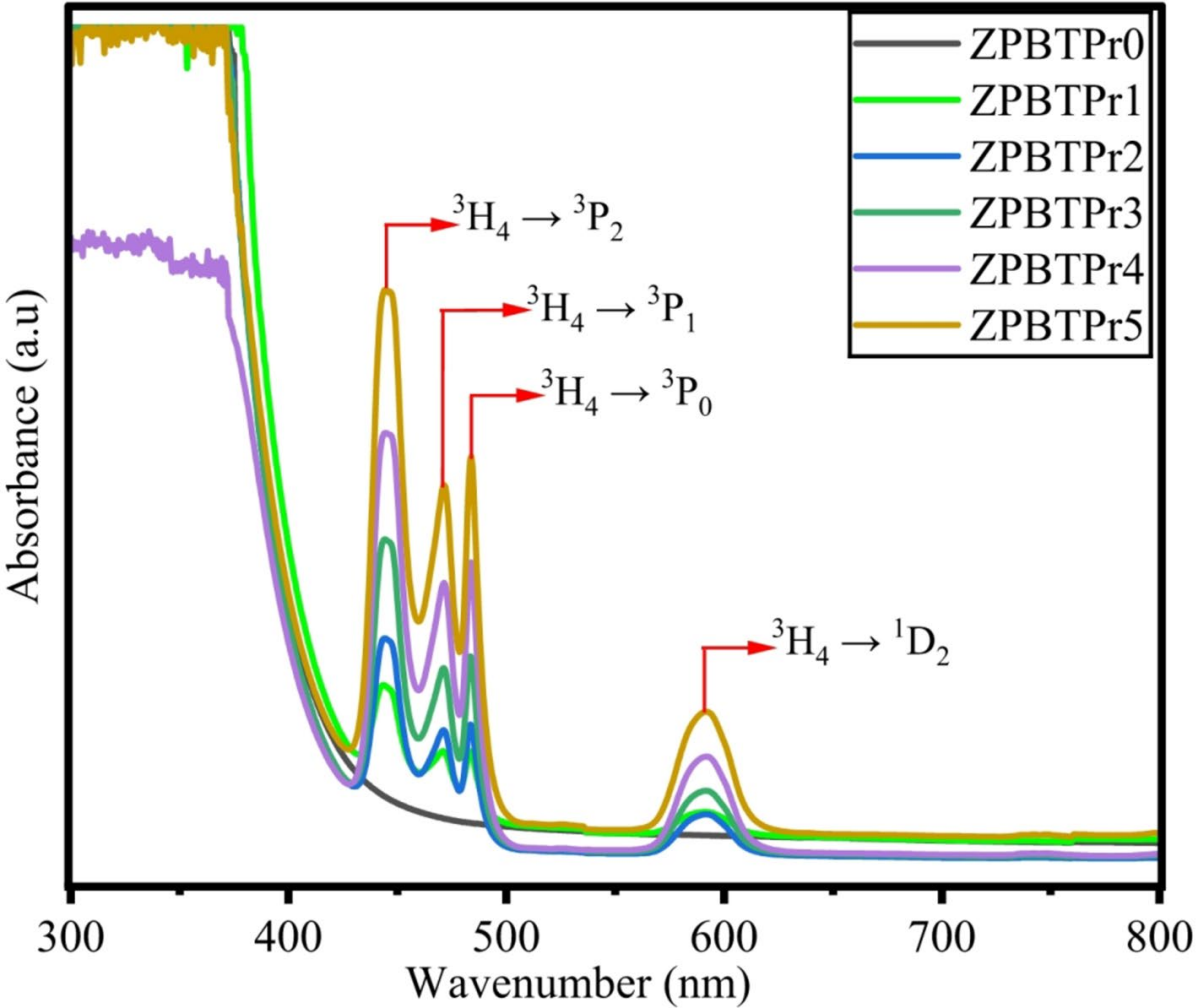
Absorption spectra of ZPBTPr glasses.

UV-Visible absorption spectra analysis serves as an effective tool, providing valuable data for determining various optical characteristics, such as the optical band gap, Urbach energy, and refractive index. The absorption spectra of ZPBTPr glasses were recorded at 300 K within the wavelength range of 300–800 nm and are shown in Fig. 12. The glass host has no absorption bands and is transparent in the measured visible range. The observed absorption peaks of Pr^3+^ ions in the doped glass samples confirm the presence of Pr^3+^ ions in the glass samples. The spectra exhibit transitions corresponding to the excitation of Pr^3+^ ions from the ground state ^3^H_4_ to various higher-energy states. The absorption peaks occurred at 444 nm, 470 nm, 485 nm and 590 nm, corresponding to ^3^H_4_ → ^3^P_2_, ^3^P_1_, ^3^P_0_, and ^1^D_2_ transitions^47^. All transitions occurred within the intra-band of 4f – 4 f. Among the observed transitions, the ^3^H_4_ to ^3^P_2_ transition is identified as hypersensitive, exhibiting a dependence on the surrounding ligand environment and governed by specific rules $\:\varDelta\:S=0;\varDelta\:L\le\:2$ and $\:\varDelta\:J\le\:2$. The absorption bands are primarily due to electric dipole transitions. The broad band at ^3^H_4_ → ^1^D_2_ is attributed to the combined effect of inhomogeneous broadening and unresolved Stark level splitting^38^. The greenish color of the glasses results from the 4f – 4f electronic transitions of Pr^3+^ ions, whereas the splitting of 4f levels produces absorption in the visible region. The coloration is also influenced by ligand field interactions surrounding the Pr^3+^ ions^21^. The determined values optical parameters of all ZPBTPr glass samples are presented in Table 5.

### Optical band gap, Urbach energy, refractive index, and steepness parameter

The optical band gap energy ($\:{\epsilon\:}^{opt}$) is a crucial parameter that offers valuable insight into the electronic structure of the glass and its effect on related optical properties like Urbach energy, refractive index, and steepness parameter, etc. The band gap can be determined using the Davis-Mott equation^27^, which is expressed as

8 $$\:\alpha\:h\vartheta\:=B{\left[h\vartheta\:-{E}^{opt}\right]}^{m}$$

Here, B $\:=$ Band tailing parameter, $\:h\vartheta\:=$ photon energy, $\:\alpha\:\left(\lambda\:\right)=2.303(A/d)$, is the absorption coefficient. d and A are the thickness and absorbance of glass samples. m specifies the kind of transition, with $\:m=$ 1/2, 2 assigned to direct and indirect permissible transitions, respectively.

Urbach energy (E_u_) can reveal the extent of structural disorder and the presence of localized energy states, within the energy band gap in non-crystalline solids. It is determined using the empirical relation^27^,

9 $$\:\mathrm{ln}\left[\alpha\:-{\alpha\:}_{0}\right]=h\vartheta\:\left[\frac{1}{{E}_{u}}\right]$$

Steepness parameter, which reflects the characteristics of the absorption edge, that results from electron-phonon interaction, was computed from the Urbach energy through the formula^85^,

10 $$\:\mathrm{S}=\:\frac{{K}_{B}T}{{E}_{u}}$$

Where, $\:{K}_{B}=$ Boltzmann constant, $\:T\:=$ Ambient temperature.

Refractive index (n) is a crucial parameter that provides valuable insights into the optical behaviour of glass materials. In the present study, the refractive index of the synthesized glass samples was evaluated utilizing Tauc’s approach in conjunction with Dimitrov and Sakka’s relation^27,85^, and is specified as

11 $$\:\frac{{n}^{2}-1}{{n}^{2}+2}=1-\:{\left[\frac{{E}_{g}}{20}\right]}^{\frac{1}{2}}$$

**Fig. 13:**
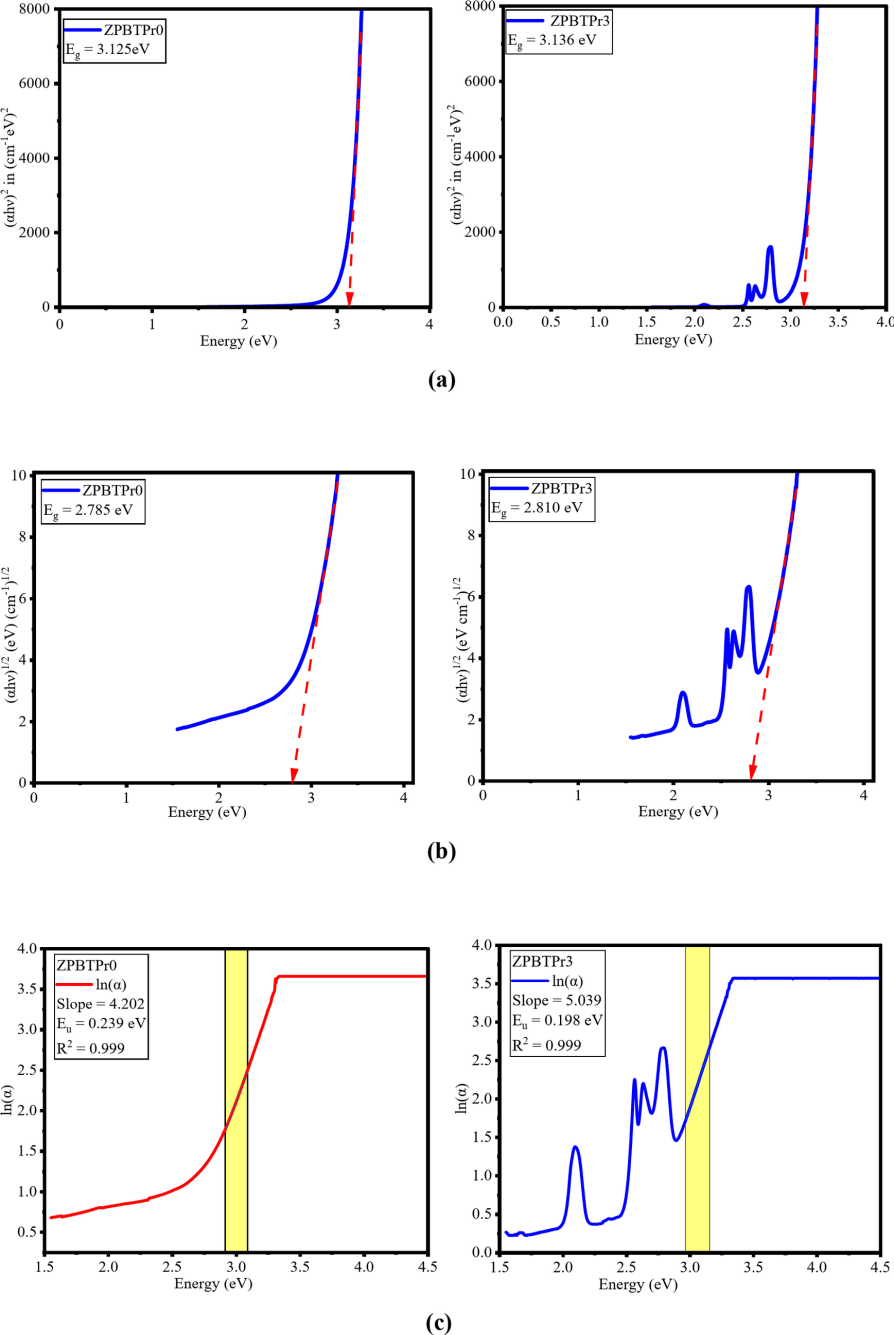
**(a)** Typical plots of h$\:\vartheta\:$ vs. $\:{\left[\alpha\:h\vartheta\:\right]}^{2}$ of ZPBTPr glasses. **(b)** Typical plots of h$\:\vartheta\:$ vs. $\:{\left[\alpha\:h\vartheta\:\right]}^{1\!\left/\:\!{2}\right.}$ of ZPBTPr glasses. **(c)** Plot of $\:h\vartheta\:\:vs\:\mathrm{ln}\propto\:$ of ZPBTPr glasses.

Optical band gaps ($\:{E}_{g}$) were determined through Tauc’s plots, and typical plots of direct and indirect energy gaps are depicted in Fig. 13**(a)** and **13(b)**. Direct ($\:{E}_{g}^{d}$) and indirect ($\:{E}_{g}^{ind}$) band gap energies of ZPBTPr glasses are tabulated in Table 5. They vary in the range of 3.154 eV – 3.110 eV and 2.833 eV – 2.748 eV, respectively. The observed trend in band gap energy is consistent with density and structural modifications. Initially, the band gap increases as Pr^3+^ is doped into the glass host. This is due to structural relaxation, as a small amount of Pr^3+^ ions occupy sites that reduce NBOs and slightly widen the optical band gap. Then, as Pr^3+^ ions increase, the energy band gap decreases slightly. This is attributed to the increase in density, formation of localized states due to structural modification by the incorporation of heavy Pr^3+^ ions by replacing lighter B-atoms. Furthermore, variations in the interionic distance and polaron radius affect electron localization, resulting in changes in the band gap energy^75^. The refractive index was found in the range of 2.356–2.368. There is an inverse correlation between n and $\:{E}_{g}$. The increase in Pr^3+^ ions content increases the structural modification by the modifiers such as PbO, and ZnO, leading to compactness and enhancement in electronic polarizability, resulting in higher n^19,23,86^. The minor variation in n is ascribed to structural modification induced by NBOs and clustering of Pr^3+^ ions, reducing the effective polarizability of the glass system. The Urbach energy (E_u_) and Steepness parameter (S) were varied in the range of 0.239–0.198 eV and 0.108–0131. The lower values of E_u_ indicate a less disordered glass system^87^. The variation of Eu is shown in Fig. 13**(C)**. S is inversely related to E_u_. Hence, E_u_ and S confirm that the optical edge becomes sharper with less disorder.

### Molar refractivity, optical dielectric constant, metallization criterion, Reflection loss, transmission coefficient, and numerical aperture

The molar refractivity $\:\left({R}_{m}\right)$, metallization criterion $\:\left({M}_{c}\right)$, and optical dielectric constant $\:\left({\epsilon\:}^{opt}\right)$ of ZPBTPr glasses are calculated by using the following relation^19,27^

12 $$\:{R}_{m}={V}_{m}\left[\frac{{n}^{2}-1}{{n}^{2}+2}\right]$$

13 $$\:{M}_{c}=1-\frac{{R}_{m}}{{V}_{m}}$$

14 $$\:{\epsilon\:}^{opt}={n}^{2}-1$$

**Fig. 14:**
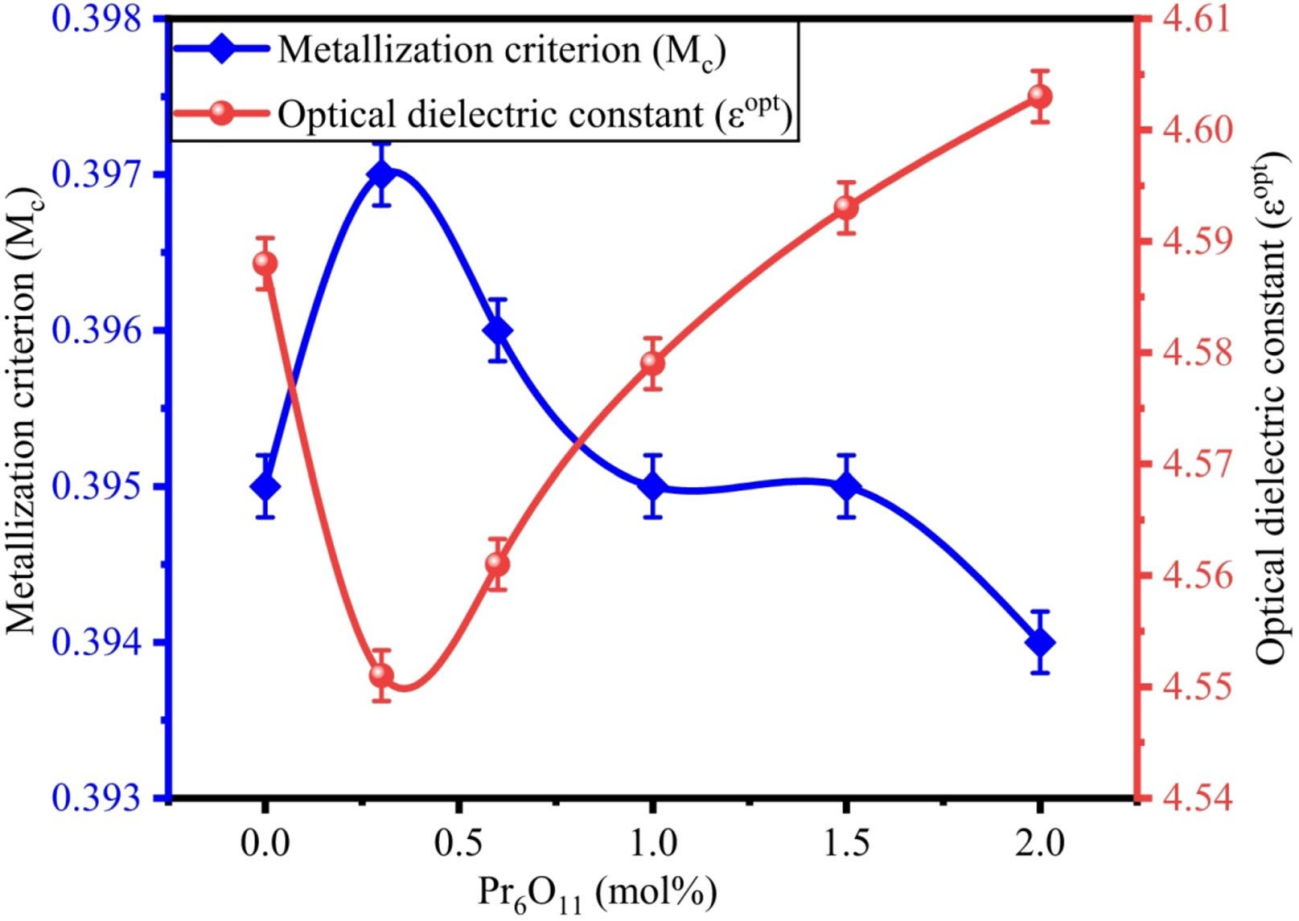
Variation of $\:{\epsilon\:}^{opt}$ vs. $\:{M}_{c}$ with Pr_6_O_11_.

The values of $\:{R}_{m}$, $\:{\epsilon\:}^{opt}$ and $\:{M}_{c}$ are listed in Table 5. The overall polarizability of a mole in a substance could be expressed as molar refractivity. It also facilitates determining molar electronic polarizability, that describes how a cloud of electrons responds to an electric field. Electronic polarizability and molar polarizability are directly proportional. The evaluated values of R_m_ vary in the range of 23.954–23.459. It is observed that there is a slight increase in R_m_ up to 1 mol%, followed by a marginal decrease and stabilization at higher concentrations of Pr^3+^. The rise in R_m_ indicates an overall increase in the electronic polarizability of the glass network due to the higher polarizability of Pr^3+^ and Pb^2+^^86^. At higher concentrations, Pr^3+^ ions do not significantly alter the electronic environment, presumably due to dopant clustering. The variations in the R_m_ show that the glass structure remains stable upon Pr^3+^ ion doping at different concentrations. The Lorentz – Lorentz relation also plays an important role in estimating the metallic nature of materials by taking the $\:{R}_{m}$ and $\:{V}_{m}$ ratio into account. According to Herzfeld’s theory of metallization, a material exhibits metallic behaviour if $\:\left(\frac{{R}_{m}}{{V}_{m}}\right)>1$, and insulating behavior if $\:\left(\frac{{R}_{m}}{{V}_{m}}\right)<1$. These parameters indicate ZPBTPr glasses have a non-metallic nature. $\:{M}_{c}$ values of ZPBTPr glasses found $\:\sim$0.39. Wang et al^86^. reported that oxides having M_c_ of 0.35–0.45 are appropriate for optical devices. Hence, ZPBTPr glasses are suitable for optical devices. The $\:{\epsilon\:}^{opt}$ values range from 4.551 to 4.603. The Pr^3+^ -ions doping causes structural modifications due to the alteration of NBOs and electronic polarizability, resulting in variations in $\:{\epsilon\:}^{opt}$^19,27^. The variations of $\:{\epsilon\:}^{opt}$ and $\:{M}_{c}$ are depicted in Fig. 14.

The reflection loss (R_L_), transmission coefficient (T), and numerical aperture (NA) of ZPBTPr glasses are evaluated by the following relations^19,27^,

15 $$\:{R}_{L}={\left[\frac{n-1}{n+1}\right]}^{2}$$

16 $$\:T=\frac{2n}{{n}^{2}+1}$$

17 $$\:NA=n\sqrt{2\times\:\varDelta\:}$$

Here, $\:n\:\mathrm{a}\mathrm{n}\mathrm{d}\:\varDelta\:$ are the refractive index, and a small change in n.

**Fig. 15:**
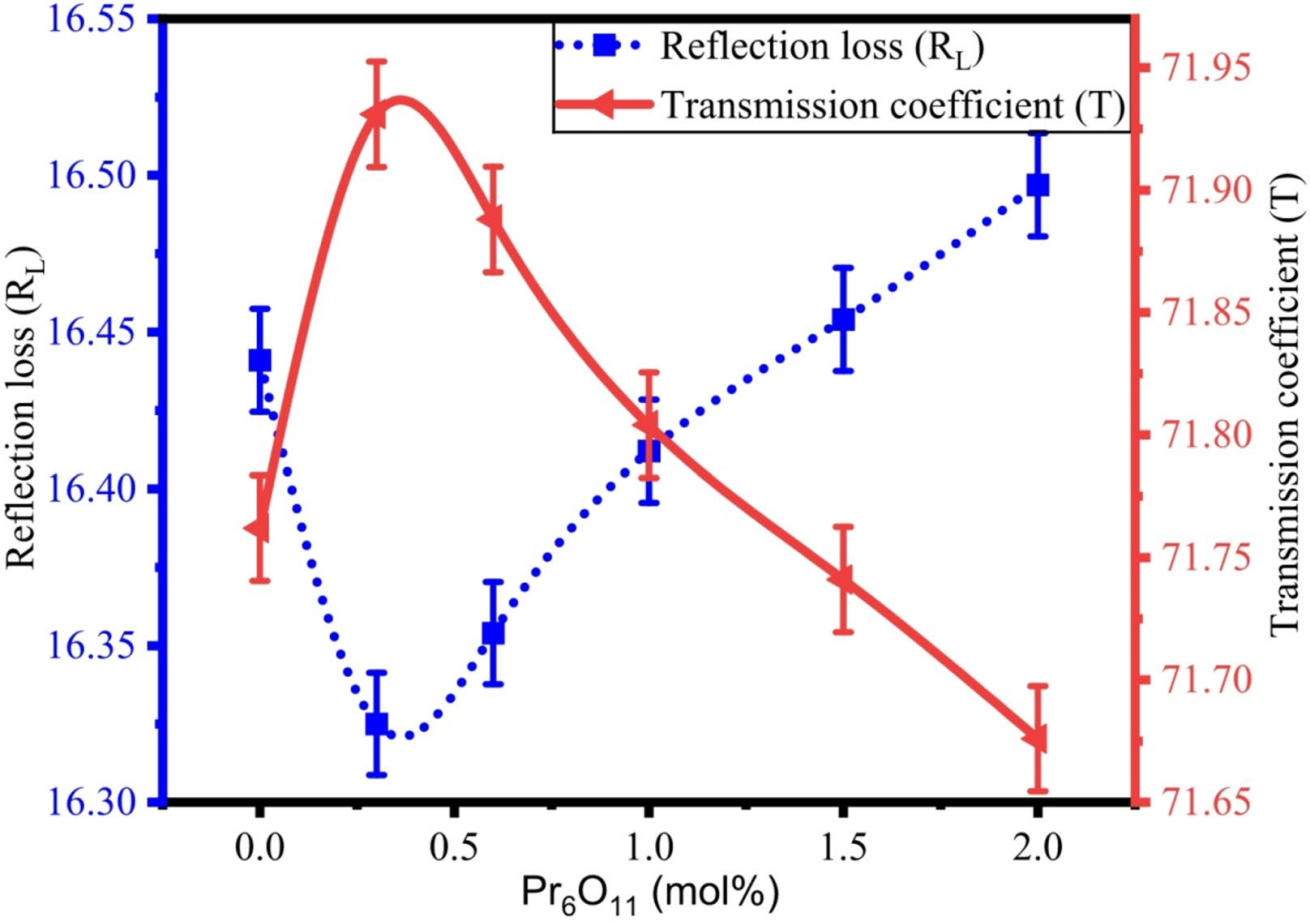
Variation of $\:{\mathrm{R}}_{\mathrm{L}}\:\mathrm{v}\mathrm{s}\:\mathrm{T}$ with Pr_6_O_11_.

The evaluated values of R_L_, T, and NA are listed in Table 5. The values of R_L_ and T found in the range of 16.325% − 16.497% and 71.931% − 71.676% respectively. The variation of R_L_ and T with praseodymium concentration is depicted in Fig. 15. The consistent values of ZPBTPr glasses retain high transparency with significant refractive index values, so Pr^3+^ ions could be introduced to provide emission centres without affecting optical performance^21^. The NA is a function of refractive index that measures a material’s ability to gather and guide light. Glasses having NA from 0.13 to 0.5 can be utilised for the core of optical fibres^88^. The variations NA are attributed to changes in n, demonstrating that the glasses hold relatively optical homogeneity and suitability for optical applications.

### Electronic oxide polarizability, electronegativity, and optical basicity

Electronic oxide polarizability ($\:{\alpha\:}_{{o}^{2-}}$), optical basicity (Λ), and electronegativity ($\:{\chi\:}_{e}$) of ZPBTPr glasses are evaluated through the following formulae^60,89,90^,

18 $$\:{\alpha\:}_{{o}^{2-}}=\frac{\left[\left(\frac{{V}_{m}}{2.52}\right)\left(\frac{{n}^{2}-1}{{n}^{2}+2}\right)-\sum\:{\alpha\:}_{cation}\right]}{{N}_{{o}^{2-}}}$$

19 $$\:\varLambda\:=1.67\left(1-\frac{1}{{\alpha\:}_{{o}^{2-}}}\right)$$

20 $$\:{\chi\:}_{e}=\frac{\varLambda\:}{0.75}+0.25$$

Where, $\:{N}_{{o}^{2-}}=$ Number of oxide ions, and $\:{\alpha\:}_{cation}$ is the molar cation polarizability.

**Fig. 16:**
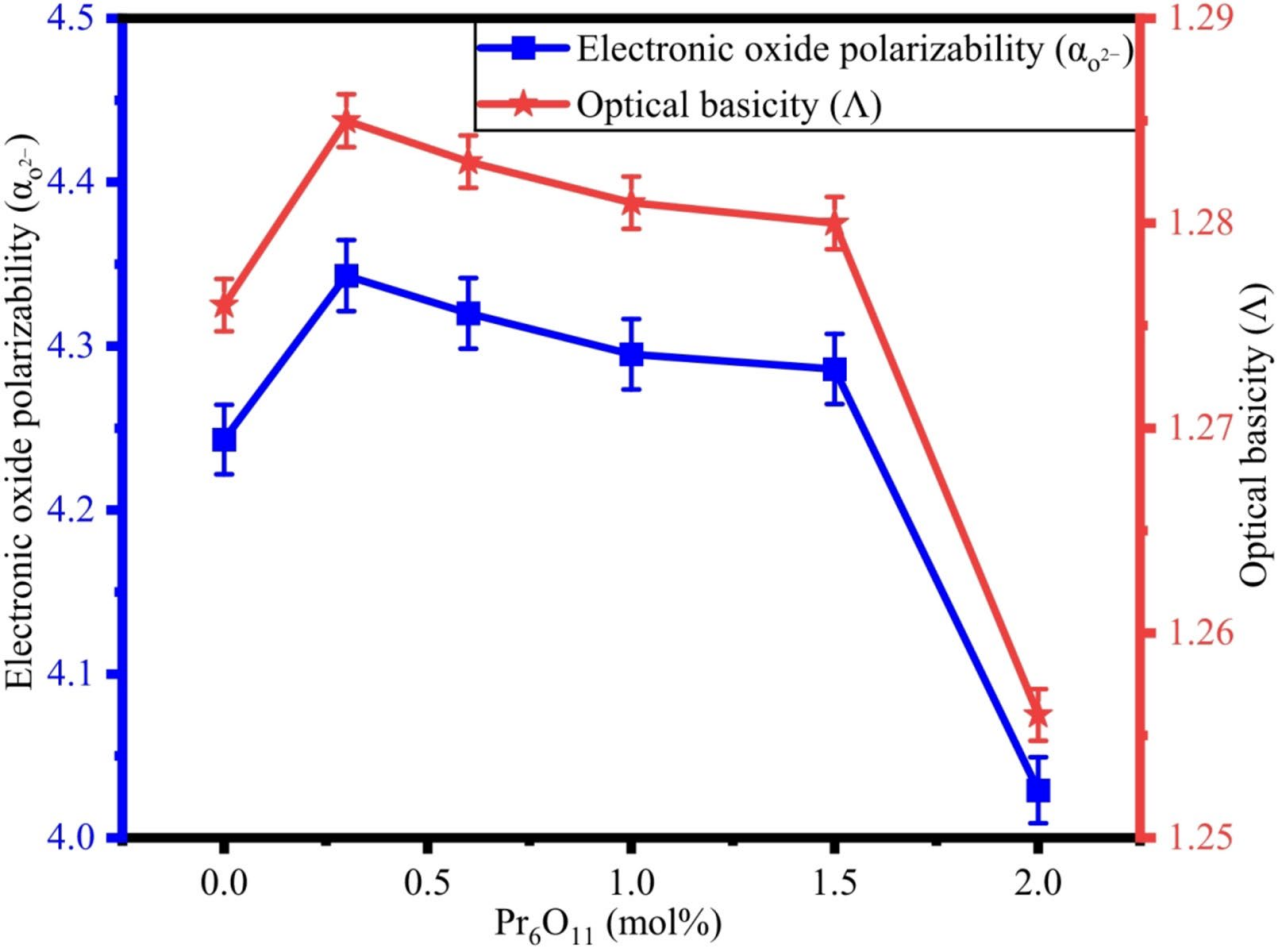
Variation of Λ vs. $\:{\alpha\:}_{{o}^{2-}}$ with Pr_6_O_11_.

Electronic oxide polarizability ($\:{\alpha\:}_{{o}^{2-}}$) represents the oxide ions’ ability in the glass network to become polarized when subjected to an external electric field. $\:{\alpha\:}_{{o}^{2-}}$ values were determined using the known values of molar polarizability^21^, $\:\left({\alpha\:}_{Te}=1.595\:\mathrm{\AA}^{3},\:{\alpha\:}_{B}=0.002\:\mathrm{\AA}^{3},\:{\alpha\:}_{Na}=0.181\mathrm{\AA}^{3},\:{\alpha\:}_{Pb}=3.623\:\mathrm{\AA}^{3},\:{\alpha\:}_{Zn}=0.283\:\mathrm{\AA}^{3},\:{\alpha\:}_{Pr}=1.38\:\mathrm{\AA}^{3}\right)$ and values of are listed in Table 5. $\:{\alpha\:}_{{o}^{2-}}$ values vary between 4.243 $\mathrm{\AA}^{3}$ and 4.376 $\mathrm{\AA}^{3}$. Initially, $\:{\alpha\:}_{{o}^{2-}}$ increases due to the inclusion of Pr^3+^ ions with larger ionic radii and high polarizing capacity, by replacing B-atoms. As Pr^3+^ increases, the $\:{\alpha\:}_{{o}^{2-}}$ slightly changes, indicating network compaction and also creation of bonds such as Pr – O – Te, Pr – O – B, that limit the mobility of ions^91,92^. The acid-base characteristic of ZPBTPr glasses is represented by their optical basicity (Λ), which reflects the electron-donating ability of oxygen within the glass. Λ varies in the range of 1.276–1.289. A similar trend has been observed, and this behavior indicates a transition from a more basic to a less basic environment or acidic nature, attributable to greater interaction of Pr^3+^ ions with O-atoms^93^. The variation of Λ and $\:{\alpha\:}_{{o}^{2-}}$ is depicted in Fig. 16. Another key factor, electronegativity ($\:{\chi\:}_{e}$) is calculated by Eq. (20), which represents the ability of atoms within the glass network to draw electrons, thereby influencing the overall bonding characteristic of glass structure. $\:{\chi\:}_{e}$ showed a correlated trend, which varies between 1.951 and 1.968. The initial rise implies an electron–attracting tendency due to the localised field effect of Pr^3+^, whereas the subsequent changes reflect a strong covalency in the glass network in relative to other oxide glasses^90,92^.

**Table 5:** Optical parameters of ZPBTPr glasses^19,27,29,60,85,89,90^. Direct band gap energy ($\:{E}_{g}^{d}$), Indirect band gap energy ($\:{E}_{g}^{ind}$), Refractive index ($\:n$), Urbach energy ($\:{E}_{u}$), Steepness parameter ($\:S$), Optical dielectric constant ($\:{\in\:}^{opt}$), Numerical aperture ($\:NA$), Molar refraction ($\:{R}_{m}$), Molar polarizability ($\:{\alpha\:}_{m}$), Electrical susceptibility ($\:\chi\:$), Metallization criterion ($\:{M}_{c}$), Reflection loss ($\:{R}_{L}$), Transmission coefficient ($\:T$), Electronic oxide polarizability ($\:{\alpha\:}_{{o}^{2-}}$), Optical basicity (Λ), Electronegativity ($\:{\chi\:}_{e}$).

Sample code	ZPBTPr0	ZPBTPr1	ZPBTPr2	ZPBTPr3	ZPBTPr4	ZPBTPr5
	$\:\pm\:$0.001	$\:\pm\:$0.001	$\:\pm\:$0.001	$\:\pm\:$0.001	$\:\pm\:$0.001	$\:\pm\:$0.001
$\:{E}_{g}^{d}$ (eV)	3.125	3.154	3.147	3.136	3.123	3.110
$\:{E}_{g}^{ind}\:\left(eV\right)$	2.785	2.833	2.821	2.810	2.751	2.748
$\:n$	2.364	2.356	2.358	2.362	2.365	2.368
$\:{E}_{u}$ (eV)	0.239	0.212	0.201	0.198	0.206	0.222
$\:S$	0.108	0.122	0.128	0.131	0.126	0.116
$\:{\epsilon\:}^{opt}$	4.588	4.551	4.561	4.579	4.593	4.603
$\:NA$	0.334	0.333	0.333	0.334	0.335	0.335
$\:{R}_{m}$	23.954	24.596	24.598	24.626	24.564	24.549
$\:{\alpha\:}_{m}$	9.505	9.760	9.761	9.772	9.747	9.742
$\:\chi\:$	0.366	0.362	0.363	0.364	0.365	0.367
$\:{M}_{c}$	0.395	0.397	0.396	0.395	0.395	0.394
$\:{R}_{L}$	16.441	16.325	16.354	16.412	16.454	16.497
$\:T$	71.762	71.931	71.888	71.804	71.741	71.676
$\:{\alpha\:}_{{o}^{2-}}\:(\mathrm{\AA}^{3}$)	4.243	4.360	4.359	4.361	4.376	4.329
Λ	1.276	1.286	1.287	1.287	1.289	1.284
$\:{\chi\:}_{e}$	1.951	1.965	1.966	1.965	1.968	1.962

## Photoluminescence studies

### Excitation spectra and emission spectra

**Fig. 17:**
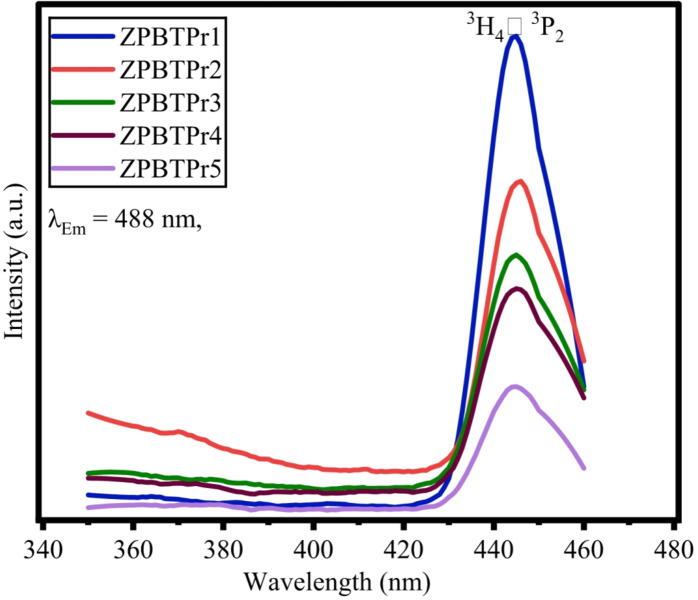
Excitation spectra of ZPBTPr glasses.

**Fig. 18:**
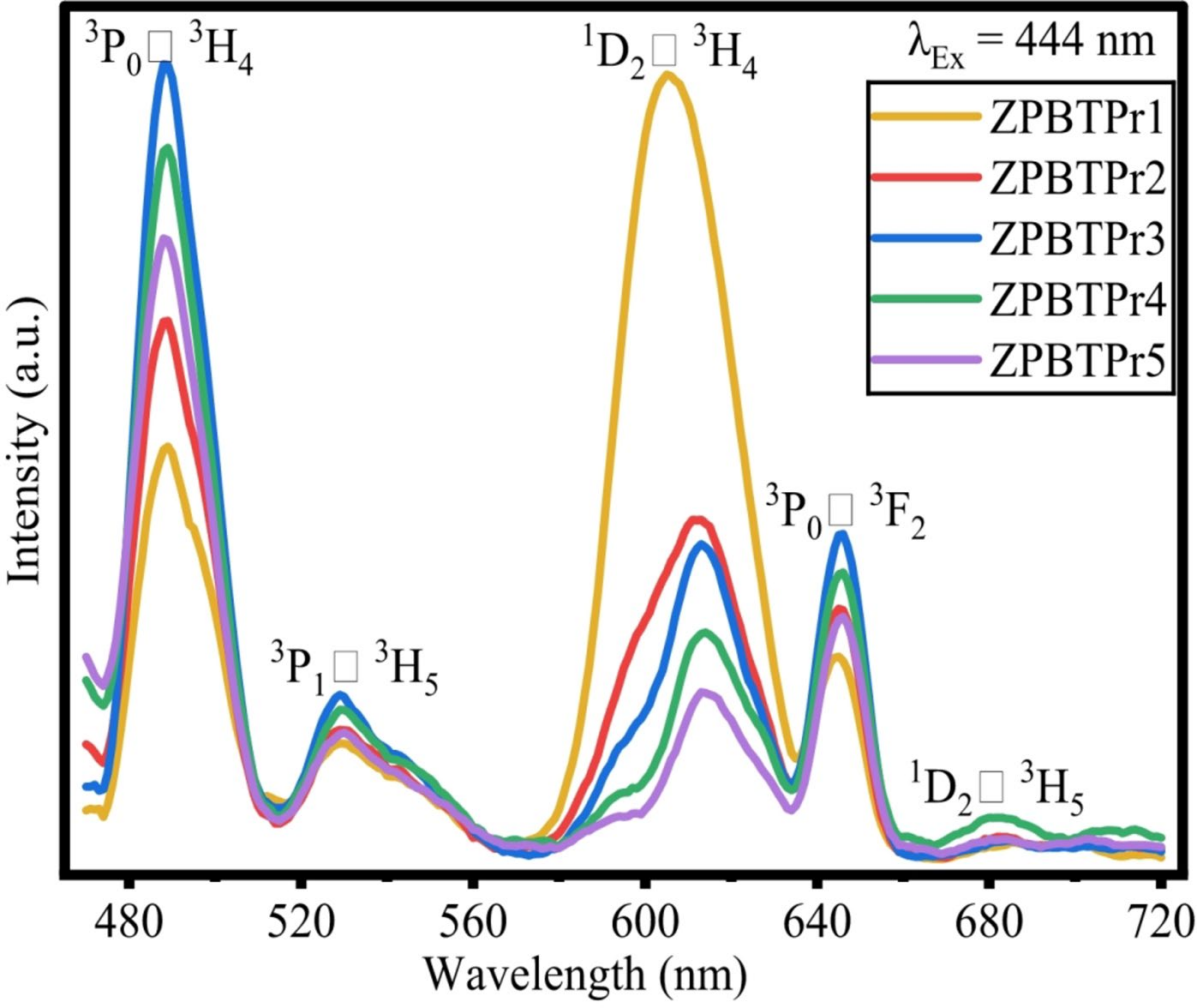
Emission spectra of ZPBTPr glasses.

The excitation spectra of ZPBTPr glasses were recorded in the range of 350–460 nm by monitoring the 488 nm wavelength, and the resulting spectra corresponding to emission at 488 nm showed high intensity at 444 nm (^3^H_4_ →^3^P_2_), is considered and depicted as shown in Fig. 17. Therefore, the luminescence spectra of the ZPBTPr glasses recorded by choosing the pumping wavelength 444 nm and the spectra is depicted in Fig. 18. The parameters considered for recording the emission spectra are Data identifier: DfltEm, Number of Data points: 291, Integration time:0.100000s, Start:460 nm, End:750 nm, Increment: 1.00 nm, Front entrance slit 1.00 nm Band pass, Front Exit slit 1.00 nm band pass, Grating: Density 1200. The emission spectra spans over the regions of blue, green, orange and red. The emission spectra exhibited transitions ^3^P_0_ → ^3^H_4_ (488 nm), ^3^P_1_ → ^3^H_5_ (528 nm), ^1^D_2_ → ^3^H_4_ (605 nm), ^3^P_0_ → ^3^F_2_ (645 nm), and ^1^D_2_ → ^3^H_5_ (680 nm), corresponding to the bands of blue, green, orange, and red^47,56,59,75,94^. The transitions ^3^P_0_ → ^3^H_4_ (488 nm, blue) and ^3^P_1_ → ^3^H_5_ (605 nm, orange) displayed higher intensities compared to the others. A pronounced suppression of the ^1^D_2_ to ^3^H_4_ (605 nm, Orange) transition is detected as the Pr^3+^ ions increase, which results in stronger blue, green, and red emissions while simultaneously weakening orange emission. In the blue band at 488 nm, the transition ^3^P_1_ to ^3^H_5_ shows an increase in intensity as the concentration of Pr^3+^ ions increases up to 1.0 mol%. The energy levels of Pr^3+^ ions are more favourably matched for blue emission, and also the phonon energy of the glass host (B_2_O_3_$\:-$TeO_2_$\:-$ZnO$\:-$PbO$\:-$Na_2_O) could assist the blue emission transitions, enhancing probabilities. Beyond this concentration, however, the luminescence quenching sets in due to the non-radiative transfer (NRT) among the populated neighboring Pr^3+^ ions in the glass. Conversely, in the orange region, at 605 nm, the transition ^1^D_2_ to ^3^H_4_ is shown to decrease as the concentration of Pr^3+^ ions increases and is found to be of high intensity at low concentration (0.3 mol%). This behavior indicates that, at low dopants, the population of the ^1^D_2_ state mainly results from rapid multi-phonon non-radiative relaxation from higher states such as ^3^P_0_, ^3^P_1_, and ^3^P_2_. At higher concentrations, cross-relaxation (CR) channels become dominant, promoting NRT and leading to quenching of luminescence, which is evident from the emission profiles^59,75,94^. At 528 nm, 645 nm, and 680 nm, the transitions give relatively lower emission. In conclusion, the variations in the intensity can thus be attributed to CR and the energy transfer process through Pr^3+^$\:-$Pr^3+^ interactions. Additionally, different transition probabilities for blue, green, and red emissions, as well as different energy state matching conditions, influence the quenching behavior.

**Fig. 19:**
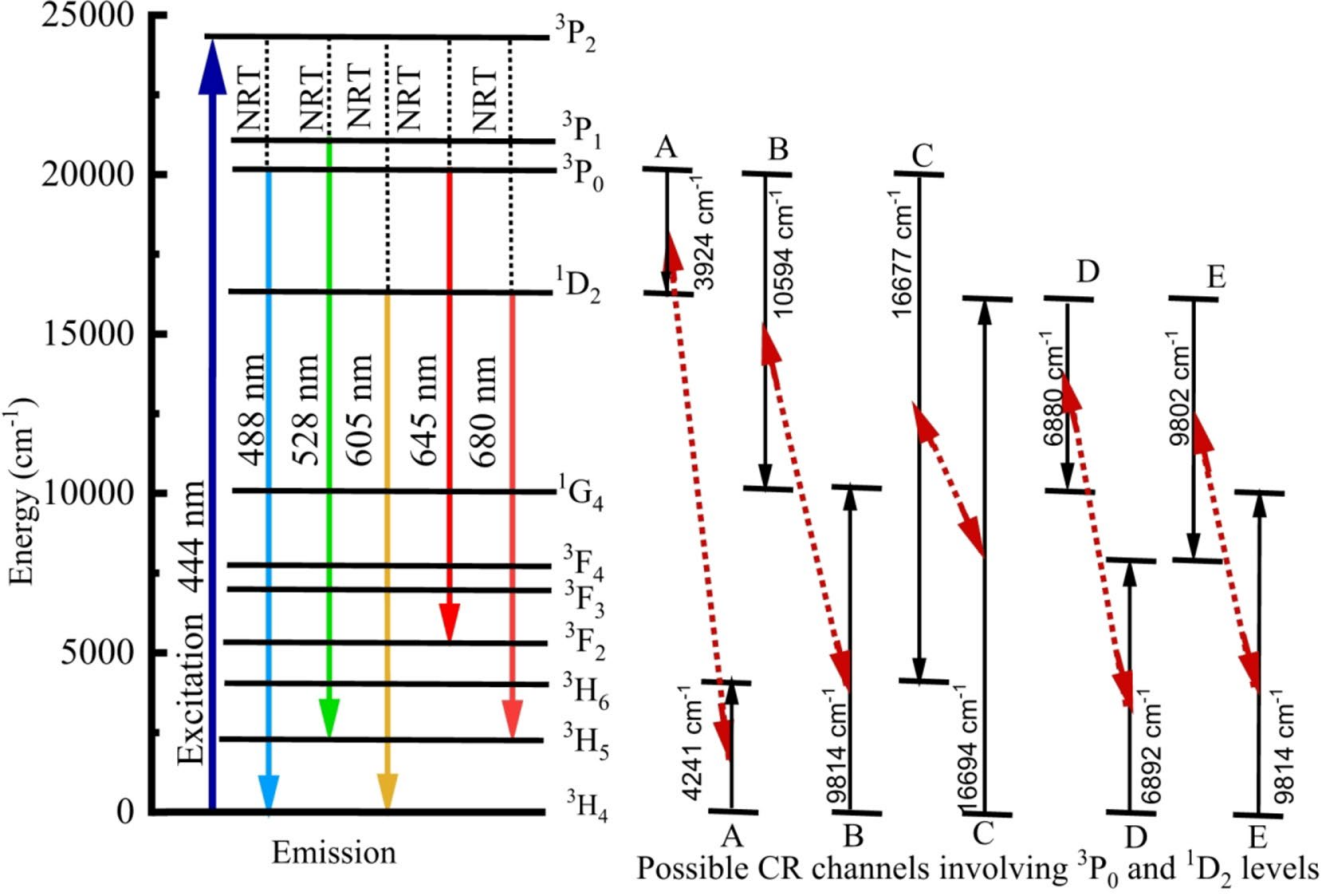
Energy level diagram of ZPBTPr glasses.

Figure 19 illustrates the partial energy diagram for the present study. Upon excitation at 444 nm, electrons undergo excitation from the ^3^H_4_ state to higher states ^3^P_2_, ^3^P_1_, ^3^P_0_, and ^1^D_2_. The excited electrons subsequently undergo non-radiative multi-phonon relaxation to lower metastable states. From these levels, radiative transitions occur to lower states, giving rise to characteristic visible emission as shown in Fig. 18. In addition, energy transfer between adjacent Pr^3+^ ions and high population at ^1^D_2_ and ^3^P_0_ through possible cross-relaxation channels influenced the emission intensity. The cross-relaxation (CR) channels involving 1D2 and 3P0 levels are as follows^95^.

A: ^1^D_2_ (i) + ^3^H_4_ (j) → ^1^G_4_ (i) + ^3^F_4_ (j).

B: ^1^D_2_ (i) + ^3^H_4_ (j) → ^3^F_4_ (i) + ^1^G_4_ (j).

C: ^3^P_0_ (i) + ^3^H_4_ (j) → ^1^D_2_ (i) + 3H_6_ (j).

D: ^3^P_0_ (i) + ^3^H_4_ (j) → ^1^G_4_ (i) + ^1^G_4_ (j).

E: ^3^P_0_ (i) + ^3^H_4_ (j) → ^3^H_6_ (i) + ^1^D_2_ (j).

Where i and j represent a pair of Pr^3+^ ions involved in the cross-relaxation process.

### CIE chromaticity diagram

**Fig. 20:**
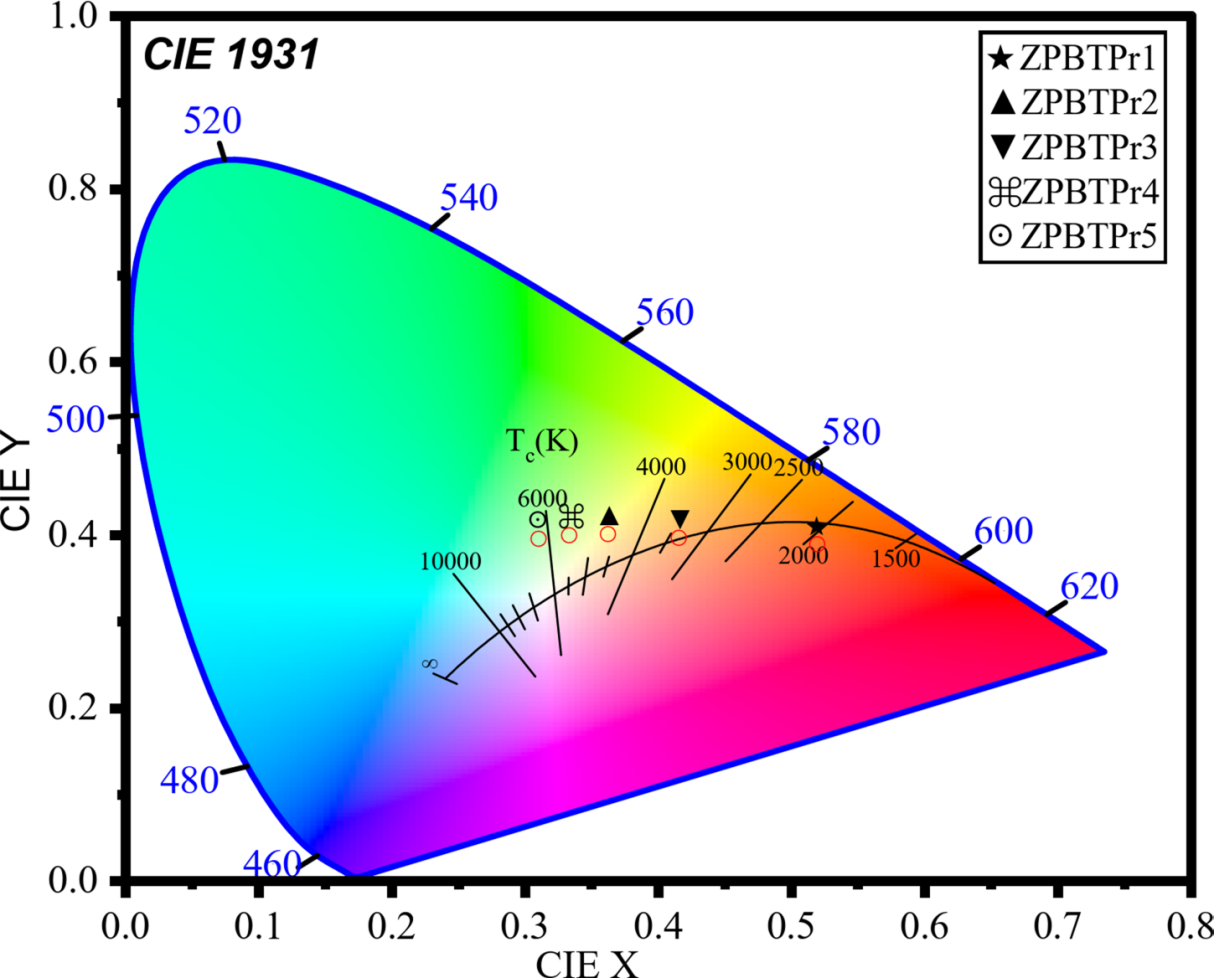
CIE diagram of ZPBTPr glasses.

The photoluminescence emission results were analysed by generating a CIE 1931 diagram, an approach by the International Commission on Illumination to assess the perceived color of light produced by the ZPBTPr glasses, and the resulting diagram is shown in Fig. 20. The location of points shows the emissions fall in the blue-green, orange, and red, highlighting their suitability for lighting and laser applications in these spectral ranges. At lower Pr^3+^ ion concentration, the transitions ^1^D_2_ → ^3^H_4_ (605 nm), and ^3^P_0_ → ^3^F_2_ (645 nm) dominate, which contribute orange-red components resulting in a warm region. At higher concentration, the ^3^P_0_ → ^3^H_4_ (488 nm) transition dominated, resulting in the cool region. The CIE coordinates shift from red to blue region is attributed to the energy transfer between Pr^3+^ ions, and population at higher energy states through the CR process. The values of (x, y)-coordinates are tabulated in Table 6. The correlated color temperature (CCT) was determined from the chromaticity coordinates (x, y) by McCamy’s approach to evaluate the light quality. CCT is a significant optical parameter that indicates the temperature of the closest to the Planckian black-body radiator corresponding to the emission point in the CIE chromaticity diagram^87^.

The McCamys’ relation is

21 $$\:CCT=\:-449{a}^{3}+3525{a}^{2}-6823a+5520.3$$

Here, $$\:a=x-0.332/y-0.186$$.

CCT values of ZPBTPr glasses were evaluated and are tabulated in Table 6. The CCT values were found from 1875.08 K to 6314.51 K. The CCT values of glass samples ZPBTPr1 and ZPBTPr2 are found to be $\:<$4000 K, and hence they are categorized under warm light sources. Whereas CCT values of glass samples ZPBTPr3, ZPBTPr4, and ZPBTPr5 are found to be $\:>$4000 K, and therefore, they can be categorized under cool light sources^87^. The findings of PL, CIE, and CCT suggest that ZPBTPr glasses have strong potential applications in the visible region for lighting and solid-state lasers, particularly more favorable in the blue-green and orange colors.

**Table 6:** CIE coordinates and CCT values.

Sample code		ZPBTPr1	ZPBTPr2	ZPBTPr3	ZPBTPr4	ZPBTPr5
(x, y)-coordinates	x	0.519	0.415	0.362	0.333	0.309
	y	0.389	0.397	0.402	0.399	0.395
CCT (K)		1875.08	3354.39	4639.44	5488.37	6314.51

## Conclusion

By the melt-quenching technique, multicomponent boro-tellurite glasses doped with Pr^3+^ ions were prepared. The XRD patterns confirm the non-crystalline nature. The structural modifications, revealed by FTIR and Raman spectroscopy, indicated the presence of functional groups BO_4_ and BO_3_, and conversion of TeO_4_ to TeO_3_ or TeO_3+1_. The SEM-EDX confirms the slight inhomogeneity and purity of the glasses, revealing the elemental composition Te, B, Zn, Pb, O, and Pr. The $\:{T}_{g}$ and $\:{T}_{c}$ vary from 409 ${\rm ^\circ C}$ to 461 ${\rm ^\circ C}$ and 511 ${\rm ^\circ C}$ to 572 ${\rm ^\circ C}$. $\:\varDelta\:T$ values are found to be above $\:100\:{\rm ^\circ C}$. This indicates the ZPBTPr glasses are thermally stable and resistant to devitrification. The density and direct energy band gap were in the ranges of 3.710 g/cm³ – 4.112 g/cm³ and 3.154 eV – 3.110 eV. The metallization criterion ($\:\sim$ 0.39), optical dielectric constant (4.551–4.603), electronic oxide polarizability (4.243 $\mathrm{\AA}^{3}$ – 4.376 $\mathrm{\AA}^{3}$), and optical basicity (1.276–1.289) values, suggesting that the obtained glasses can be contemplated for non-linear optical devices. Electronic transitions of Pr^3+^ ions, viz. ^3^P_0_ → ^3^H_4_ and ^3^P_1_ → ^3^H_5_, exhibit intense blue and orange emissions corresponding to 488 nm and 605 nm wavelengths, respectively. The CCT value of the ZPBTPr1, which exhibits intense orange emission, is $\:<$ 4000 K. The CCT value of ZPBTPr3, which exhibits intense blue emission, is $\:>$ 4000 K. Based on the structural modifications induced by Pr^3+^ ions, as well as the thermal and luminescence features, along with the efficacy of optical and physical parameters, the obtained ZPBTPr glasses are promising materials for luminescent devices and solid-state lasers.

## Data Availability

The data used and/or analysed during the current study are available from the corresponding author on reasonable request.
